# ERRα promotes glycolytic metabolism and targets the NLRP3/caspase-1/GSDMD pathway to regulate pyroptosis in endometrial cancer

**DOI:** 10.1186/s13046-023-02834-7

**Published:** 2023-10-20

**Authors:** Pingping Su, Xiaodan Mao, Jincheng Ma, Lixiang Huang, Lirui Yu, Shuting Tang, Mingzhi Zhuang, Zhonglei Lu, Kelvin Stefan Osafo, Yuan Ren, Xinrui Wang, Xite Lin, Leyi Huang, Xiaoli Huang, Elena Ioana Braicu, Jalid Sehouli, Pengming Sun

**Affiliations:** 1 Laboratory of Gynecologic Oncology, Department of Gynecology, College of Clinical Medicine for Obstetrics & Gynecology and Pediatrics, Fujian Maternity and Child Health Hospital, Fujian Medical University, Fuzhou, 350001 Fujian China; 2 Women and Children’s Hospital, School of Medicine, Xiamen University, Xiamen, China; 3 Fujian Key Laboratory of Women and Children’s Critical Diseases Research, Fujian Maternity and Child Health Hospital, Fuzhou, 350001 Fujian China; 4 Fujian Clinical Research Center for Gynecologic Oncology, Fujian Maternity and Child Health Hospital, Fujian Obstetrics and Gynecology Hospital, Fuzhou, 350001 Fujian China; 5 College of Biological Science and Engineering, Fuzhou University, Fuzhou, 350108 Fujian China; 6 Medical Research Center, College of Clinical Medicine for Obstetrics and Gynecology and Pediatrics, Fujian Maternity and Child Health Hospital, Fujian Medical University, Fuzhou, 350001 Fujian China; 7 NHC Key Laboratory of Technical Evaluation of Fertility Regulation for Non-Human Primate, Fujian Maternity and Child Health Hospital, Fuzhou, 350001 Fujian China; 8 Department of Obstetrics and Gynecology, The First Affiliated Hospital, Fujian Medical University, FuzhouFujian, 350005 China; 9 Department of Gynecology and Obstetrics, Charité Virchow University Hospital, Augustenberger Platz1, 13353 Berlin, Germany; 10 National Key Clinical Specialty Construction Program of China (Gynecology), Fujian Maternity and Child Health Hospital, Fuzhou, 350001 Fujian China

**Keywords:** Endometrial cancer, Pyroptosis, Metabolic reprogramming, ERRα, Cisplatin resistance

## Abstract

**Background:**

Tumor cells can resist chemotherapy-induced pyroptosis through glycolytic reprogramming. Estrogen-related receptor alpha (ERRα) is a central regulator of cellular energy metabolism associated with poor cancer prognosis. Herein, we refine the oncogenic role of ERRα in the pyroptosis pathway and glycolytic metabolism.

**Methods:**

The interaction between ERRα and HIF-1α was verified using co-immunoprecipitation. The transcriptional binding sites of ERRα and NLRP3 were confirmed using dual-luciferase reporter assay and cleavage under targets and tagmentation (CUT&Tag). Flow cytometry, transmission electron microscopy, scanning electron microscopy, cell mito stress test, and extracellular acidification rate analysis were performed to investigate the effects of ERRα on the pyroptosis pathway and glycolytic metabolism. The results of these experiments were further confirmed in endometrial cancer (EC)-derived organoids and nude mice. In addition, the expression of ERRα-related pyroptosis genes was analyzed using The Cancer Genome Atlas and Gene Expression Omnibus database.

**Results:**

Triggered by a hypoxic microenvironment, highly expressed ERRα could bind to the promoter of NLRP3 and inhibit caspase-1/GSDMD signaling, which reduced inflammasome activation and increased pyroptosis resistance, thereby resulting in the resistance of cancer cells to cisplatin. Moreover, ERRα activated glycolytic rate-limiting enzyme to bridge glycolytic metabolism and pyroptosis in EC. This phenomenon was further confirmed in EC-derived organoids and nude mice. CUT & Tag sequencing and The Cancer Genome Atlas database analysis showed that ERRα participated in glycolysis and programmed cell death, which resulted in EC progression.

**Conclusions:**

ERRα inhibits pyroptosis in an NLRP3-dependent manner and induces glycolytic metabolism, resulting in cisplatin resistance in EC cells.

**Supplementary Information:**

The online version contains supplementary material available at 10.1186/s13046-023-02834-7.

## Background

Tumor cells easily form a local hypoxic microenvironment owing to their rapid growth, resulting in increased expression of hypoxia-inducible factor-1 alpha (HIF-1α) [1]. *HIF-1α*, a key gene among hypoxia-induced genes, participates in the metabolic reprogramming of cancer cells in hypoxic and nutrient-deficient environments [2]. Under conditions of limited oxygen, tumor cells favor the aerobic glycolytic pathway to meet their energy requirements, which is known as metabolic reprogramming (the Warburg effect) [3]. This process provides energy to tumor cells and raw materials for the synthesis of nucleotides, lipids, and other substances required for cell proliferation [4]. Tumor cells that adapt to the hypoxic microenvironment through metabolic reprogramming can avoid the lethality of anti-cancer drugs and maintain survival, which may result in chemotherapy resistance [5, 6]. Moreover, treatment with platinum induces cytotoxic death and programmed cell death in tumors, including apoptosis and pyroptosis [7].

Research has focused on tumor resistance to cellular apoptosis and pyroptosis, especially on anti-pyroptotic cells [8]. Pyroptosis is a form of pro-inflammatory programmed cell death closely associated with tumor cell proliferation, invasion, and metastasis and can influence the outcome of chemotherapy for tumors [9]. Pyroptosis is characterized by cell swelling, plasma membrane rupture, large bubble formation on the cell membrane, and pro-inflammatory cytokine release. NLRP3/caspase-1/GSDMD is a classical pyroptosis pathway. NLRP3 stimulation leads to inflammasome formation and caspase-1 activation. Subsequently, pro-IL-1β and pro-IL-18 are converted to their active forms, the N-terminus of GSDMD is transferred to the cell membrane, and the cell membrane is ruptured, resulting in the release of cell contents and induction of cell pyroptosis [10, 11]. The caspase-3/GSDME pathway is a non-classical pyroptosis pathway. However, the activation of the non-classical pyroptosis pathway does not depend on the activation of inflammasomes [12]. Pyroptosis resistance can inhibit antitumor immunity and promote the development of multiple types of cancer [13]. Tumor cell resistance to pyroptosis can induce chemotherapy resistance, whereas the promotion of cell pyroptosis can enhance the clinical effect of anticancer therapies in patients with tumors [14]. Platinum is one of the most commonly used antitumor chemotherapeutic drugs for the treatment of gynecologic cancers [15]. However, platinum resistance is a common phenomenon and results in cancer recurrence and metastasis, both of which remain major challenges in clinical practice. It is unclear whether increased cellular resistance to pyroptosis would result in the resistance of gynecologic tumors to platinum.

We previously reported that the overexpression of estrogen-related receptor alpha (ERRα) promotes endometrial cancer (EC) invasion and metastasis [16]. EC is one of the most common malignant tumors of the female reproductive tract [17]. Although substantial progress has been made in EC treatment, 7%–15% of patients with early-stage EC (stages I–II) experience tumor recurrence [18]. Moreover, the disease-free recurrence rate within 5 years for patients with advanced (stages III–IV A) disease is 58%–59% [19], and the 5-year survival rate remains below 20% [20]. ERRα is a nuclear transcription factor and central regulator of energy metabolism. High ERRα expression indicates a more aggressive and malignant phenotype, as reflected in chemotherapy resistance and tumor metastasis, which are closely related to poor prognosis [21]. We reviewed the possible mechanisms through which HIF-1α/ERRα regulates the expression of metabolism-related genes and various signaling pathways in malignant tumors, thereby regulating the energy metabolism of tumor cells and resistance to pyroptosis [22]. Furthermore, ERRα is a potential tumor-promoting factor in EC. However, the specific mechanism through which ERRα induces cisplatin (known as DDP) resistance in EC requires further clarification. We hypothesize that surviving cancer cells with a high ERRα expression could promote metabolic reprogramming of EC cells, prevent pyroptosis, and induce DDP resistance. EC recurrence and mortality could be reduced by exploring the mechanisms underlying EC drug resistance, identifying new genes that are potential biomarkers of platinum chemosensitivity, and developing targeted drugs to improve the efficacy of chemotherapy, thereby improving treatment outcomes in patients.

## Methods

### Cell lines and cell culture

Human KLE and HEC-1A endometrial carcinoma cells (KeyGEN BioTech Co., Ltd., Jiangsu, China) were cultured in PRMI-1640 and McCoy’s 5A Medium independently, both containing 1% penicillin–streptomycin(#15,140,122, Gibco™) and 10% fetal bovine serum (FBS) (#10,091,148, Gibco, New Zealand) at 37℃ in moist atmosphere (5% CO_2_, 95% air). All cell lines were verified by short tandem repeat (STR) DNA profiling analysis. Lentiviral vectors LV-sh-ESRRA (named siERRα) was synthesized by Genechem (Shanghai, China). The siRNA target sequence in the ERRα (LV-ESRRA-RNAi, 55,367–1) was 5’-TACCACTATGGTGTGGCAT-3’. The lentiviral vector used in overexpressing ERRα were named ovERRα. Negative control was purchased from Genechem (Shanghai, China). We silenced ERRα expression (siERRα) and its empty vector (used as a control) by the specific shRNA lentiviral vector, and overexpressed ERRα vector and its empty vector (used as an overexpression control), which were labeled by EGFP, then transfected them all into stable clones. Overexpression of ERRα was achieved in KLE and HEC-1A cells and named KLE^−ovERRα^ and HEC-1A^−ovERRα^. In addition, KLE and HEC-1A cells with ERRα down regulation were named KLE^−siERRα^ and HEC-1A^−siERRα^.

### Cell counting kit-8

The cell suspension was prepared and inoculated into a 96-well plate with six repetitive wells. After adhering to the wall, different concentrations of drugs were added to each well and cultured in the incubator at 37℃ for a corresponding time. 10ul CCK-8 enhanced solution (MA0218, meilunbio, Dalian Meilun Biotechnology Co.,Ltd) was added to each well subsequently. Incubate in the incubator for 0.5–4 h, and measure the absorbance of 450 nm.

### Hoechst 33,342/PI

KLE and HEC-1A cells were inoculated in a 6-well plate. After drug treatment, it was washed with PBS, and then 0.8-1 ml cell staining buffer was added (CA1120, Solarbio, Beijing Solarbio Science & Technology Co., Ltd). 5ul Hoechst staining solution and 5ul PI staining solution were added subsequently, mixed and incubated at 4℃ for 20–30 min. After staining, it was washed once with PBS and then observed under a fluorescence microscope.

### Flow cytometry detection

The cells were digested with trypsin without EDTA and collected by centrifugation. After adding 1 × binding buffer working solution to the cell precipitation, the cell suspension (the total number of cells was 1 × 10^5^cell) was added to a new tube. 5 μl AnnexinV-PE and 10 μl 7-AAD were added, mixed gently and incubated at room temperature in the dark for 15 min. Flow cytometry detection was performed on the computer.

### Transmission electron microscopy

Collect cells precipitate, re-suspended and fixed for 2–4 h at 4℃ with TEM fixative. After centrifugation, the supernatant was discarded and 0.1 M phosphate buffer PB (pH 7.4) was added. 1% agarose solution was prepared, and the precipitate was suspended and wrapped in the agarose. After 1% OsO4 was fixed at room temperature for two hours, it was dehydrated with ethanol. Resin penetration and embedding are done with acetone and Embed812 in the right proportion and kept in a 37℃ oven overnight. After being polymerized at 65℃ for 48 h, the resin blocks were cut to 60-80 nm and stained, observed under a transmission electron microscope, and the images were collected and analyzed.

### Lactate dehydrogenase

Substrate, chromogenic agent and coenzyme were used to prepare the reaction working solution in proportion (#E-BC-K766-M, Elabscience Biotechnology Co., Ltd). Add 50ul of standard substances with different concentrations into the corresponding standard wells of enzyme-labeled plates, then take 50ul of supernatant of cells to be detected, and add them into the corresponding measurement wells of enzyme-labeled plates. 50ul reaction working solution was added to each well and incubated for 10 min at 37℃. After incubation, a 50ul stop solution was added to each hole. The OD value of each well was measured at 450 nm.

### Western blotting

Standard techniques were used for protein quantification, separation, transfer, and blotting in KLE and HEC-1A cells. Primary antibodies immunoblotting antibodies were used: ERRα (1: 500, ab76228, Abcam, London, UK), ERRα (1:500, E1G1J, Cell Signaling Technology, Massachusetts, USA), NLRP3 (1:500; 19,771-1AP, Proteintech, Wuhan, China), HIF-1α (1:500; 20,960-1AP, Proteintech, Wuhan, China), cleaved GSDMD (1:500; #36,425, Cell Signaling Technology, Massachusetts, USA), GSDMD (1:500, E9S1X, Cell Signaling Technology, Massachusetts, USA), GSDME (1:500, 84005S, Cell Signaling Technology, Massachusetts, USA), Caspase1 (22,915–1-AP, Proteintech, Wuhan, China), Caspase-1 (YT5743, Immunoway, USA), Caspase-1 (1:500, D7F10, Cell Signaling Technology, Massachusetts, USA), Caspase-3 (1:500, D3R6Y, Cell Signaling Technology, Massachusetts, USA), IL-18 (1:500,10,663–1-AP, Proteintech, Wuhan, China), cleaved-IL-1β (1:500, D3A3Z, Cell Signaling Technology, Massachusetts, USA), β-actin (1:2000, YM3028l, Immunoway, USA), β-tublin (1:2000, YM3030, Immunoway, USA), and GAPDH (1:2000; YM3029, Immunoway, USA).

### Luciferase reporter assays

Various target genes of NLRP3 were retrieved from the (UCSC) database (https://genome.ucsc.edu/cgi-bin/hgTracks?db=hg38&lastVirtModeType=default&lastVirtModeExtraState=&virtModeType=default&virtMode=0&nonVirtPosition=&position=chr1%3A247414077%2D247416176&hgsid=1488170781_6nweGbKLWMfAOch4ZenEAlVZpk2A). We found that ESRRA (estrogen-related receptor alpha) can transcribe and bind to the promoter region of *NLRP3*, as the Supplement Fig. 1 shown. The JASPAR database (https://jaspar.genereg.net/analysis) was used to identify the potential binding sites of the NLRP3 promoter region of ERRα. The NLRP3 promoter sequence (247,414,077 bp to 247,416,176 bp) (Supplement Fig. 2) relative to the transcription start site was amplified by PCR and inserted into the pGL3-basic plasmid. Three *NLRP3* promoter fragments with gradually decreasing lengths were ligated to the pGL3-basic plasmid: NLRP3-1 (-400 to + 21), NLRP3-2 (-800 to + 21), and NLRP3-3 (-1200 to + 21). The potential sequence 3′-ACAACTTGAACACGGAAACG-5′ was mutated and named NLRP3-2-MUT. 293 T cells were cotransfected with ERRα transcription factor, NLRP3 promoter labeled with luciferase reporter, and renilla luciferase in 24-well plates with lipofectamine 3000 (L3000075, INVITROGEN, USA). After 48 h, the firefly and renilla luciferase activities were measured using the Dual-Lite Luciferase Assay System (DD1205, Vazyme, Nanjing, China) by a microplate reader (Gen1), and the ratio of firefly/renilla luciferase activity was calculated.

### RT-qPCR

Lyse cells directly in a culture dish by adding RNA Extraction. Vortex the cell lysate thoroughly. Centrifuge the samples and transfer the supernatant to a new tube. Add 0.25 ml of chloroform per 1 ml of RNA Extraction. Following centrifugation, transfer the upper aqueous phase into a fresh tube. Precipitate the RNA by mixing it with isopropyl alcohol. Wash RNA with 75% ethanol. First Strand cDNA Synthesis and preparation of PCR Master Mix (#A2791, Promega, Madison, USA). PCR amplification was performed using Eastep QPCR Master Mix (#LS2062, Promega, Madison, USA). Relative gene expression was calculated using the 2^−ΔΔCt^ method, with GAPDH as the reference gene.

### Cell mito stress test

The oxygen consumption rate (OCR) was conducted using Seahorse XF Cell Mito Stress Test Kit (#103,015–100, Agilent Technologies, USA). SeahorseXF basic medium (#103,575–100, Agilent Technologies, USA) was added with 1 mM pyruvate, 2 mM glutamine and 10 mM glucose as the detection solution. Oligomycin, FCCP and Rot/AA were prepared correctly into a working solution, which was correspondingly added into the dosing hole on the probe board. The cell growth medium was replaced by a warm detection solution, and the cell culture microplate was placed in a CO_2_-free incubator at 37℃ for an hour. SeaHorseXF24 (Agilent Technologies, USA) was then run on the computer and the data were analyzed.

### Glycolysis stress test

The glycolysis stress test was conducted by using Seahorse XF Glycolysis Stress Test Kit (#103,020–100, Agilent Technologies, USA). SeahorseXF basic medium (#103,334–100, Agilent Technologies, USA) was added with 2 mmol glutamine as the detection solution. Oligomycin, glucose and 2-DG were properly prepared into a working solution, which was correspondingly added into the dosing hole on the probe board. The cell growth medium was replaced by a warm detection solution, and the cell culture microplate was placed in a CO_2_-free incubator at 37℃ for an hour. SeaHorseXF24 (Agilent Technologies, USA) was then run on the computer and the data were analyzed.

### Co-immunoprecipitation

HEC-1A^−ovERRα^ cells were harvested in 10 cm culture and then added with IP buffer for cell lysis (containing protease inhibitor 1: 100), lysed on ice for 40 min, and the cell lysate was centrifuged at 4℃. One cell lysate was reserved as the input group for WB analysis, and the remaining three were incubated overnight with ERRα antibody (1-2ug; sc-65720, Santa Cruz Biotechnology), HIF-1α antibody (0.5–4.0ug; 20,960-1AP, Proteintech, Wuhan, China) and IgG antibody (1.0ug; 36113ES10, Yeasen, Biotechnology), respectively. After pretreatment, protein A agarose beads were added into the cell lysate incubated with the antibody overnight for 2–4 h to couple the antibody with protein A agarose beads; After immunoprecipitation, agarose beads were isolated and detected by SDS-PAGE and WB.

### Double immunofluorescence staining

The cell climbing slides were permeabilized, blocked, and incubated with the mixed reagent of the first and second primary antibodies, and then, the corresponding secondary antibody was added. DAPI solution was dripped into the circle and incubated, then coverslip with anti-fade mounting medium. Microscopy detection and collect images by Fluorescent Microscopy.

### Scanning electron microscopy

Seed cells on a sterile cover glass in a petri dish, followed by adding electron microscopy fixative into petri dish. Post-fix, dehydrate, and drying. Specimens are attached to metallic stubs using carbon stickers and sputter-coated with gold for 30 s. Observe and take images with scanning electron microscope.

### Cut&Tag for cell samples, library preparation, sequencing and data analysis

Cleavage under targets and tagmentation (CUT&Tag) (N259-YH01, Novoprotein, Jiangsu, China) assay was performed as the standard techniques. Briefly, the cells are bound to concanavalin A-coated magnetic beads, and the cell membrane is permeabilized by digitonin. The enzyme pA-Tn5 transposase precisely binds the DNA sequence near the target protein under the antibody guidance and results in factor-targeted tagmentation. The DNA sequence is tagmented, with adapters added simultaneously at both ends, which PCR enriches to form the sequencing-ready libraries. After the PCR reaction, libraries were purified with the AMPure beads and library quality was assessed on the Agilent Bioanalyzer 2100 system. After sequencing and quality control, the reads were mapped to the reference genome. ChIPseeker was used to retrieve the nearest genes around the peak and annotate the genomic region of the peak. ChIPseeker can confirm peak-related genes.

### Immunohistochemical staining

Immunohistochemical staining was performed according to standard procedures. Rabbit polyclonal anti-ERRα (1:100; #ab137489, Abcam, London, UK), cleaved GSDMD (1:100; #36,425, Cell Signaling Technology, Massachusetts, USA), and NLRP3 (1:100; 19,771-1AP, Proteintech, Wuhan, China) antibodies were used. Three low-power visual fields (× 100) were randomly selected under the microscope, and the corresponding scores of strong staining, moderate staining, weak staining, and negative were 3, 2, 1 and 0 according to the positive staining intensity. According to the proportion of stained cells, they were divided into 76% ~ 100%, 51% ~ 75%, 26% ~ 50%, and 0 ~ 25% and the corresponding scores were 4, 3, 2 and 1 points. The multiplication of the intensity and percentage of positively stained cells is the immunohistochemical score of the sample. The Samples were divided into four groups based on their immunoreactive scores: 0, negative (-);1–4, weakly positive ( +); 5–8, positive (+ +); and 9–12, strongly positive (+ + +).

### Organoid

Fresh tumor tissues from patients with endometrial cancer were obtained by operation, cut and digested, resuspended with matrigel matrix and plated, and cultured in a 3D organ-like medium containing various cytokines for three days. The number of different organoids tested in the study is shown in the Supplement Table 1. The growth and morphology of organs were observed and photographed under microscope. The working concentration of the drug: DDP 20 μmol/L and XCT790 10 μmol/L. The drug was dissolved with 0.9% NaCl_2_ or dimethyl sulfoxide (DMSO), and the control group was added with the same volume of 0.9% NaCl_2_ or DMSO. After three days of drug treatment, samples were collected for follow-up detection.

### In vivo mice xenograft assay

Female BALB/c nude mice (*N* = 20, age 4–5 weeks) were purchased from Zhejiang Weitong Lihua Experimental Animal Technology Co., Ltd. (Charles river). The KLE cell line and ERRα overexpression cell line (KLE^−ovERRα^) were transplanted into the forelimb armpit of female BALB/c mice. Three weeks after tumor cell transplantation, the mice were equally divided into four groups: the KLE group (Vehicle-KLE xenograft mice treated with DMSO as negative control), the KLE^+DDP^ treatment group (10 mg/kg DDP was injected intraperitoneally into the xenograft mice once per week for 28 days), the KLE^+DDP+XCT790^ treatment group (5 mg/kg XCT790 was injected intraperitoneally into the xenograft mice every three days for 28 days), and the KLE^−ovERRα+DDP^ treatment group (10 mg/kg DDP was injected intraperitoneally into the xenograft mice once per week for 28 days). The tumor size was measured every three days, and the tumor volume was calculated using the following formula: tumor volume = (length × width × width) / 2. At the end of the study, the mice were anesthetized and killed. Carefully remove tumor tissue and other organs, including liver, spleen, kidney and heart, for further research.

### Bioinformatics data analysis

ESRRA, pyroptosis-related gene expression and clinical data of GTEx were obtained from the UCSC Xena database (https://xenabrowser.net/datapages/). The clinical data of the EC cohort came from the data of TCGA (https://cancergenome.nih.gov/). The expression of RNA-Seq gene FPKM (genes per kilobase per million) and clinical data of 587 EC patients were preserved and analyzed. In addition, corresponding information in the GSE17025 dataset of patients with EC was collected from the GEO (Gene Expression Omnibus) database. The RNAseq data in FPKM format is converted to TPM (transcripts per million reads) format and converted to log2 for further analysis. According to the median expression level of ESRRA in TCGA EC samples, patients were divided into low-expression group and high-expression groups. GO database and KEGG access database were used for analysis. The functions of GO genes are divided into three categories: biological process (BP), cellular component (CC) and molecular function (MF). Using the GO database, we can get the relationship between the target genes at CC, MF and BP levels. KEGG is a collection of databases used to systematically analyze gene functions and associate related gene sets with their pathways. In this study, we studied the functions of ESRRA and its co-expressed genes. Then, we select a subset of representative terms from this cluster to visualize and set the p-value to 0.05. The cluster profiler package of R software is used for statistical analysis and visualization.

### Statistical analysis

SPSS 23.0 software (IBM SPSS, Armonk, NY, USA) was used for statistical analysis, and GraphPad Prism 8 and ImageJ software packages were used for drawing. The experiments were repeated at least three times. Data were calculated as (mean ± SD). Student’s t- test was used in western blot, cell proliferation, Hoechst33342/PI staining, flow cytometry analysis and IHC score of protein expression. P < 0.05 was considered statistically significant.

## Results

### Pyroptosis and NLRP3/caspase-1/GSDMD pathway are activated in EC cells treated with DDP

**Fig. 1:**
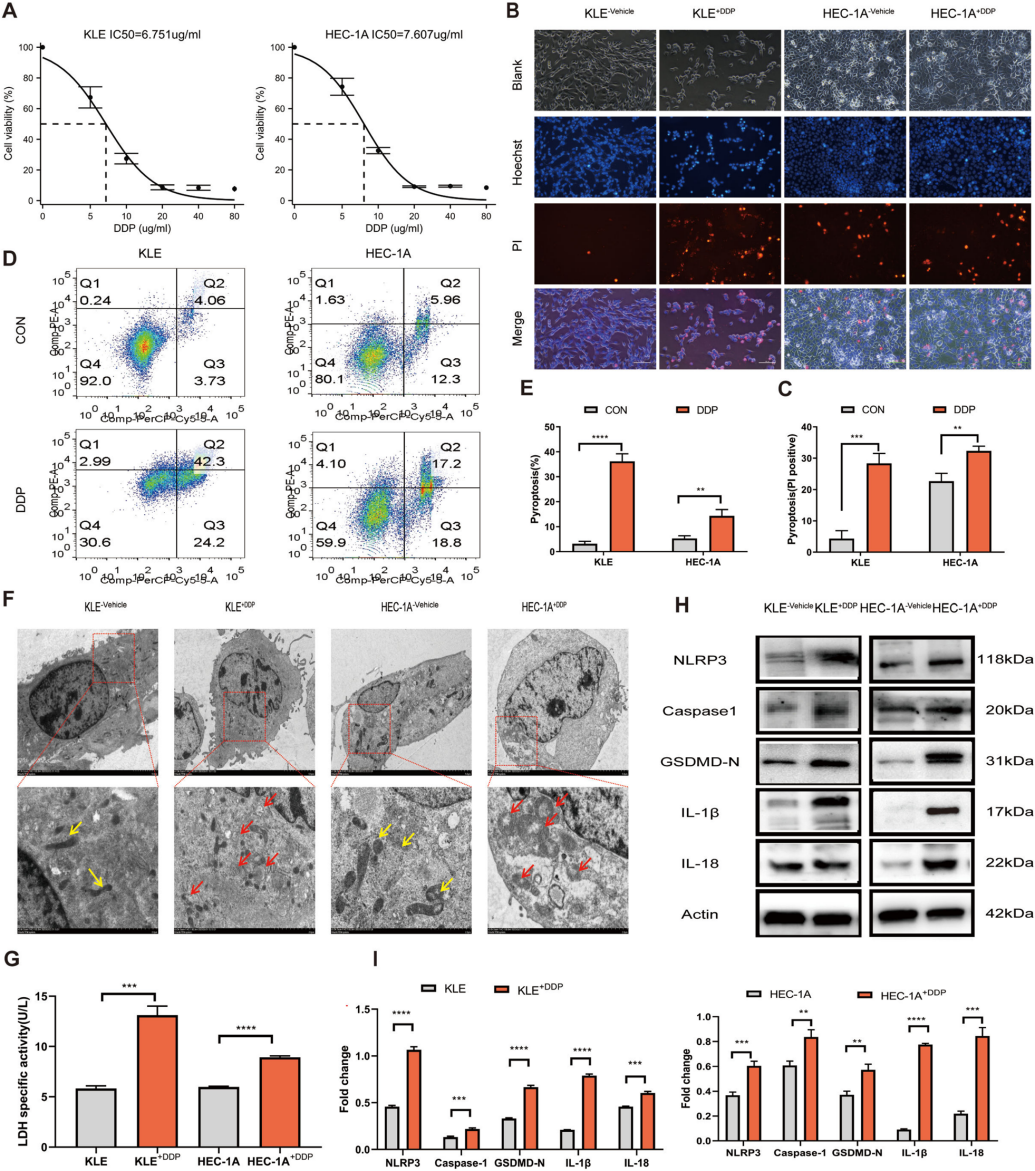
Pyroptosis is induced via the NLRP3/caspase-1/GSDMD pathway when KLE and HEC-1A cells are treated with DDP. **A** KLE and HEC-1A cells were treated with DDP at various concentration gradients (0, 5, 10, 20, 40, and 80 µg/mL) for 48 h; then, cell viability was assessed using the CCK8 assay. **B**, **C** The pyroptotic cells (PI-positive) were imaged using fluorescence microscopy after DDP treatment for 12 h (the values in each graph represent the average of three random fields per sample); scale bar: 100 µm. The KLE and HEC-1A cells from the control group were treated with the vehicle 0.9% NaCl. **D**, **E** The percentages of pyroptotic Annexin V-PE- and 7-AAD-positive cells within the second quadrant (Q2) were determined using flow cytometry after treating KLE and HEC-1A cells with DDP for 24 h. CON indicates the control group of KLE and HEC-1A cells treated with the vehicle 0.9% NaCl. **F** Representative TEM images of KLE and HEC-1A cells treated with DDP for 12 h. The mitochondrial membrane was disrupted, the matrix was dissolved, and the mitochondrial cristae were broken, as indicated with red arrows. The control group KLE and HEC-1A cells were treated with the vehicle 0.9% NaCl; the yellow arrows indicate the mitochondria structures that were more complete. Scale bar: 2 μm. **G** LDH activity in the culture supernatants of KLE and HEC-1A cells treated with DDP for 12 h was measured. **H**, **I** Whole-cell lysates of KLE and HEC-1A cells treated with DDP for 12 h or left untreated were analyzed using WB. The control group KLE^−vehicle^ and HEC-1A^−vehicle^ cells were treated with 0.9% NaCl. β-Actin was included as a loading control. The dose of DDP in the above-mentioned in vitro experiments was 7 µg. Data are representative of three independent experiments and shown as the mean ± SD. *p < 0.05; **p < 0. 01; ***p < 0.001; ****p < 0.0001. Abbreviations: CCK8, Cell Counting Kit-8; PI, propidium iodide; TEM, transmission electron microscopy; LDH, lactate dehydrogenase; WB, western blotting

Platinum is the first-line chemotherapeutic drug for the treatment of EC. Initially, the appropriate dosage for in vitro cell experiments was chosen by determining the 50% inhibitory concentration (IC_50_) of DDP in KLE and HEC-1A cells using the Cell Counting Kit-8 assay (CCK8) (Fig. 1A). DDP treatment for 48 h significantly decreased the viability of KLE and HEC-1A cells in a dose-dependent manner. The IC_50_ of DDP was approximately 6.751 and 7.607 μg/mL in KLE and HEC-1A cells, respectively. Subsequently, the effects of DDP on the growth and pyroptosis of EC cells in vitro were determined using DDP at 7 μg/mL. Following DDP treatment, pyroptosis of EC cells was observed (Fig. 1B–I). Hoechst33342 and propidium iodide (PI) stain pyroptotic cells as these stains can permeate ruptured membranes, causing strong red and blue fluorescence, respectively. Hoechst33342/PI double staining showed that the number of pyroptotic KLE and HEC-1A cells was higher in the group treated with DDP for 12 h than in the control group (Fig. 1B-C). Annexin V-PE/7AAD double staining was used for flow cytometry analysis; the percentage of Annexin V-PE/7AAD double-positive pyroptotic cells (second quadrant) increased significantly after DDP treatment compared with that in the control group (Fig. 1D-E). Consistent with these results, an increase in lactate dehydrogenase (LDH) release was observed after DDP treatment, indicating that DDP disrupted the membrane integrity of KLE and HEC-1A cells (Fig. 1G). Transmission electron microscopy (TEM) of EC cells showed that after DDP treatment, the mitochondrial membrane was disrupted, matrix was dissolved, and mitochondrial cristae were broken (Fig. 1F). Therefore, DDP may disturb energy metabolism in KLE and HEC-1A cells and activate the pyroptosis pathway. The levels of caspase-1, IL-18, and IL-1β increased in KLE and HEC-1A cells after treatment with DDP for 12 h compared with the those in the controls, as determined using western blot (WB) analysis, and NLRP3 inflammasomes were subsequently activated. The level of the N-terminal cleavage product of GSDMD (GSDMD-N) was upregulated compared with that in the controls (Fig. 1H-I). Therefore, pyroptosis and NLRP3/caspase-1/GSDMD pathway activation were observed in EC cells treated with DDP.

### ERRα binds to the promoter of *NLRP3* and inhibits the downstream caspase-1/GSDMD pathways

**Fig. 2:**
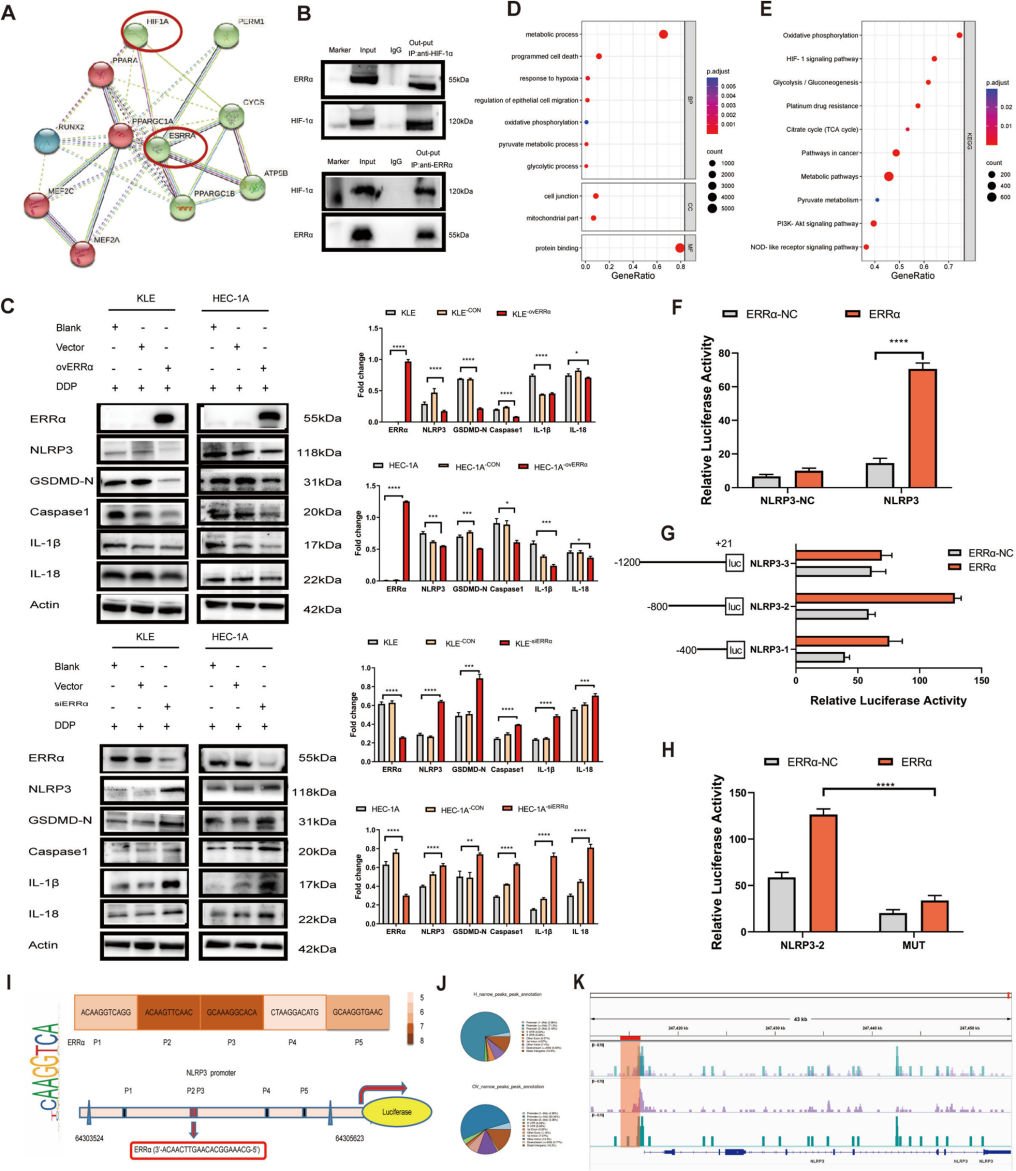
ERRα inhibits caspase-1/GSDMD signaling via transcriptional binding with *NLRP3*. **A** Interaction between HIF-1α and ERRα was found in the STRING protein interaction database. **B** Interaction between HIF-1α and ERRα was verified using the co-immunoprecipitation (Co-IP) analysis of HEC-1A^−ovERRα^ cell lysates. The corresponding antibodies (out-put IP: anti-HIF-1α-antibody and anti-ERRα-antibody) could precipitate both ERRα and HIF-1α, respectively, in the HEC-1A^−ovERRα^ cell lysates. **C** Effects of ERRα regulation on the levels of pyroptosis-related proteins (NLRP3, GSDMD-N, caspase-1, IL-1β, and IL-18) in EC cells was analyzed using WB; β-actin was used as the loading control. Untreated KLE and HEC-1A cells and the empty vector-transfected KLE and HEC-1A cells treated with DDP were used as the control groups. **D** GO analysis showing the enriched biological process in EC cells overexpressing ERRα, HEC-1A cells were used as the control group. **E** KEGG pathway analysis for the genes that showed ERRα-binding peaks in their promoter regions. **F**–**H** Dual-luciferase reporter assay to assess the interaction between ERRα and NLRP3. (F) 293 T cells were co-transfected with ERRα transcription factor, NLRP3 promoter labeled with luciferase reporter, and the *Renilla* luciferase control. The negative-control (NC) group was NLRP3-NC with ERRα-NC and NLRP3 with ERRα-NC. (G) Three NLRP3 promoter fragments ligated to the pGL3-basic plasmid, named NLRP3-1 (-400 to + 21), NLRP3-2 (-800 to + 21), and NLRP3-3 (-1200 to + 21), were co-transfected with ERRα transcription factor; the three NLRP3 promoter fragments with ERRα-NC were used as the control. (H) NLRP3-2-MUT and wild-type NLRP3-2 and their empty vectors were co-transfected with ERRα transcription factor. The vector-control group was NLRP3-2 with ERRα-NC and MUT with ERRα-NC. The luciferase activity was measured and normalized to that of the *Renilla* luciferase control 48 h post-transfection and the relative luciferase activity was determined. **I** Schematic diagram of putative ERRα-binding sites, as predicted by the online program JASPAR, located in the NLRP3 promoter region (P1–P5). The sequence TCAAGGTCA of ERRα is placed on the left. **J** CUT&Tag and sequencing analysis of the distribution map of ERRα peaks in gene functional regions. **K** *NLRP3* promoter occupancy in HEC-1A cells was evaluated. ERRα was immunoprecipitated using an anti-Flag antibody. Data are representative of three independent experiments and shown as the mean ± SD. *p < 0.05; **p < 0. 01; ***p < 0.001; ****p < 0.0001. Abbreviations: ERRα, estrogen-related receptor alpha; HIF-1α, hypoxia-inducible factor-1 alpha. Co-IP, co-immunoprecipitation. CON; control empty vector. NC: negative control. MUT: mutation

Hypoxic microenvironments created by the rapid growth of tumor cells can promote HIF-1α expression. Our findings from the STRING protein interaction database (Fig. 2A) verified the interaction between HIF-1α and ERRα through co-immunoprecipitation (Fig. 2B). The corresponding antibodies precipitated both ERRα and HIF-1α but did not precipitate the negative control IgG protein. To further study the crosstalk between ERRα and HIF-1α, CUT&Tag assay was performed, and the sequencing results confirmed that ERRα could directly bind to the promoter of *HIF-1α* (Supplement Fig. 3 and Supplement Table 2). To verify the effect of HIF-1α expression on the upregulated expression of ERRα in EC cells under hypoxia, KLE and HEC-1A cells were treated with CoCl_2_, a hypoxic simulator, for 24 h. After 12 h of treatment, 200 µm CoCl_2_ upregulated the expression of HIF-1α and ERRα in KLE and HEC-1A cells compared with that in cells under normal oxygen conditions (Supplement Fig. 4A). However, the expression of HIF-1α was not affected by *ERRα* knockdown or overexpression (Supplement Fig. 4B). This result confirmed that HIF-1α is the upstream regulator of ERRα. When a cell undergoes pyroptosis, the N-terminus of GSDMD is transferred to the cell membrane, and the cell membrane is ruptured by drilling [23]. To determine whether the interaction between ERRα and HIF-1α regulates downstream signals, we then quantified the proteins involved in the pyroptosis signaling pathway to determine the mechanism through which ERRα regulates pyroptosis in EC cells treated with DDP (Fig. 2C). After 12 h of DDP treatment, pyroptotic inflammasomes were activated, accompanied by an increase in the level of NLRP3 and the cleavage of caspase-1, which promoted the expression of GSDMD-N, IL-18, and IL-1β proteins. Upregulation of ERRα inhibited the expression of the pyroptosis-related proteins NLRP3, GSDMD-N, caspase-1, IL-18, and IL-1β, whilst downregulation of ERRα upregulated the levels of these proteins. Regulation of ERRα expression does not lead to significant changes in the caspase-3/GSDME pathway (Supplement Fig. 5). We then analyzed the interaction and functional combination between ERRα and corresponding target genes in EC cells overexpressing ERRα, with the HEC-1A cells were used as the control group. GO analysis revealed that most of the enriched biological processes were related to the response to hypoxia, programmed cell death, and glycolytic process. Most importantly, KEGG pathway analysis showed that the HIF-1 signaling, platinum drug resistance, nod-like receptor signaling, glycolysis, metabolic, and other pathways were enriched (Fig. 2D-E).This finding confirmed that high expression of ERRα could inhibit EC cell pyroptosis.

We then retrieved various target genes of NLRP3 from the University of California Santa Cruz (UCSC) database to analyze the molecular mechanisms involved in tumor pyroptosis. *NLRP3*, a classic marker gene that controls pyroptosis, was identified as a potential target gene of ERRα (Supplement Fig. 1). Dual-luciferase reporter analysis was conducted to determine whether ERRα can trigger the activation of the *NLRP3* promoter. The result showed that co-transfection of *NLRP3* and *ERRα* significantly increased luciferase activity, compared with that in the vector-control group of 293 T cells (Fig. 2F). This finding confirmed the functional interaction between ERRα and the *NLRP3* promoter. To further identify the transcription binding site of ERRα on the *NLRP3* promoter, we measured the activity of the potential promoter and determined the minimum sequence required for the activity using different truncate and mutant designs. Compared with that of NLRP3-1 and NLRP3-3, the fluorescence activity of NLRP3-2 increased significantly (Fig. 2G), indicating that there may be one cis-acting element in the -800 to -400 region that can be bound to *NLRP3*. An analysis tool of the JASPAR database was used to construct mutants in the -800 to -400 region and named NLRP3-2-MUT. The luciferase activity was then determined, as shown in Fig. 2H. Compared with that of NLRP3-2, the fluorescence activity of NLRP3-2-MUT decreased significantly, confirming the existence of the binding site of the transcription factor ERRα in NLRP3. Based on whether ERRα regulates the expression of pyroptosis-related proteins in EC cells, we concluded that the overexpression of ERRα mediates pyroptosis inhibition in EC cells by regulating the expression of NLRP3 at the transcriptional level. Furthermore, the site with the 3′-ACAACTTGAACACGGAAACG-5′ sequence was most likely to bind to ERRα (Fig. 2I). To further study the crosstalk between ERRα and NLRP3, CUT&Tag assay was performed, and the sequencing results showed that ERRα-related peaks were distributed in gene functional regions; moreover, ERRα could directly bind to the promoter of *NLRP3* (Fig. 2J-K; Supplement Table 2). These findings strongly indicate that ERRα directly binds to the *NLRP3* promoter and negatively regulates the expression of NLRP3.

### Overexpression of ERRα enhances pyroptosis resistance and induces EC cell resistance to DDP

**Fig. 3:**
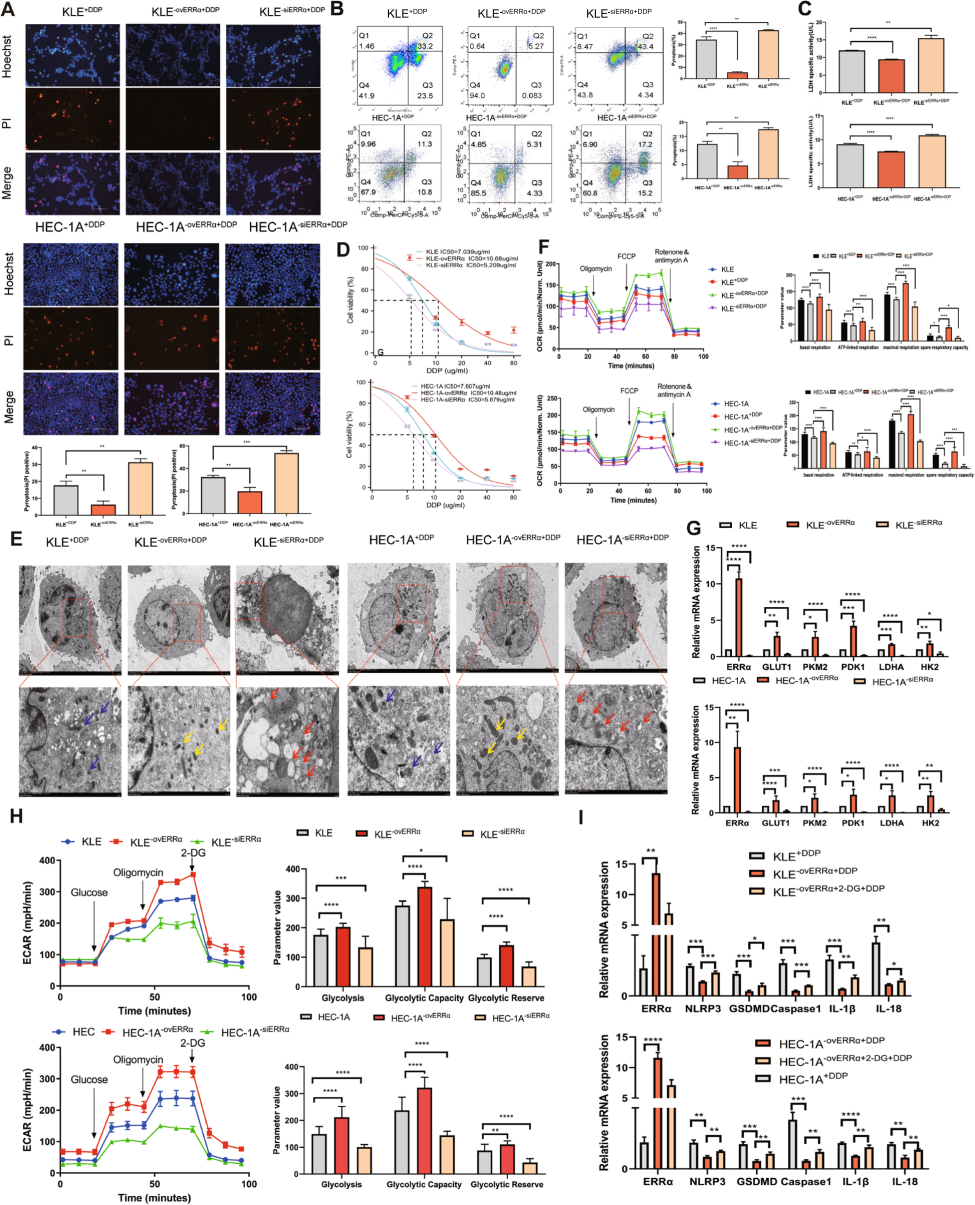
Overexpression of estrogen-related receptor alpha (ERRα) enhances pyroptosis resistance accompanied by glycolytic metabolism, leading to cisplatin (DDP) resistance of EC cells. **A** Pyroptotic cells (PI-positive) in each well were imaged using fluorescence microscopy after DDP treatment for 12 h in the ovERRα and siERRα groups. KLE and HEC-1A cells treated with DDP for 12 h were included as the control group in the experiment. The values in each graph represent the average of three random fields per sample. Scale: 100 µm. **B** Percentage of AnnexinV-PE- and 7AAD-positive pyroptotic cells within the second quadrant (Q2) from different ERRα expression groups was analyzed using flow cytometry after 24 h of DDP treatment. KLE and HEC-1A cells treated with DDP for 24 h were used as the control group. **C** LDH activity of cell culture supernatants was measured in different ERRα expression groups after DDP treatment for 12 h. KLE and HEC-1A cells treated with DDP for 12 h were included as the control group in the experiment. **D** KLE and HEC-1A cells were treated with DDP at various concentration gradients for 48 h, and the IC_50_ of DDP in different ERRα expression groups was determined using the CCK8 assay. **E** Representative TEM images of KLE and HEC-1A cells in different ERRα expression groups treated with 7 µg DDP for 12 h. The mitochondrial membrane structure was indistinctly dissolved, the matrix was dissolved in a large area, and the crest was broken in siERRα cells, as indicated with the red arrows; the yellow arrows indicate ERRα-overexpressing EC cells whose mitochondrial membrane structure was relatively clear, the matrix was partially dissolved, and the cristae were slightly broken. The blue arrows indicate the mitochondria in the KLE and HEC-1A cells of control group treated with DDP for 12 h. Scale: 2 μm. **F** Basal, ATP-linked, and maximal respiration and spare respiratory capacities were assessed to evaluate the mitochondrial function in different ERRα expression groups. KLE^+DDP^ and HEC-1A^+DDP^ cells were used as the control groups. **G** Total RNA in KLE and HEC-1A cells was assessed using RT-qPCR to measure the expression of glycolysis-related genes in the ovERRα and siERRα groups. KLE and HEC-1A cells were used as the control group. **H** Extracellular acidification rate, glycolysis, glycolytic capacity, and glycolytic reserve in different ERRα expression groups are shown. KLE and HEC-1A cells were used as the control group. **I** Total RNA in KLE and HEC-1A cells was assessed using RT-qPCR to measure the expression of pyroptosis-related genes in EC cells. KLE^−ovERRα+DDP^ and HEC-1A^−ovERRα+DDP^ cells were used as the control groups. The results are presented as the average of three experimental replicates. Data are shown as the mean ± SD. Statistical tests: Student’s* t*-test. *p < 0.05; **p < 0.01; ***p < 0.001; ****p < 0.0001. Abbreviations: EC, endometrial cancer; IC_50_, inhibitory concentration; PI, propidium iodide; TEM, transmission electron microscopy; 2-DG, 2-deoxy-glucose

To determine the role of ERRα in the NLRP3/caspase-1/GSDMD pathway, we used Hoechst33342/PI double staining to quantify cell viability (Fig. 3A). We observed that after 12 h of DDP treatment, the proportion of cells undergoing pyroptosis was lower in the ERRα-overexpression group, whereas, it was higher in the *ERRα*-knockdown EC cell group than in the HEC-1A and KLE cells. Flow cytometry analysis showed that the percentage of AnnexinV-PE- and 7AAD-positive pyroptotic cells was 5.27% and 5.31% in KLE^−ovERRα+DDP^ and HEC-1A^−ovERRα+DDP^, respectively, whereas it was 43.4% and 17.2% in KLE^−siERRα+DDP^ and HEC-1A^−siERRα+DDP^, respectively (Fig. 3B). Interestingly, upregulation of ERRα inhibited DDP-induced tumor cell pyroptosis, whereas downregulation of ERRα promoted DDP-induced cell apoptosis, consistent with the above findings. The LDH assay (Fig. 3C) showed that the level of LDH released by EC cells overexpressing ERRα was lower than that in the blank group, whereas the level of LDH released by *ERRα*-knockdown EC cells increased compared with that in HEC-1A and KLE cells. This finding implies that the integrity of the cell membrane can also be affected by ERRα regulation. We then verified the effect of different levels of ERRα expression on the sensitivity of cells to DDP. The IC_50_ for EC cells overexpressing ERRα was 10.48–10.68 µg/mL, and that for the siERRα cells was 5.209–5.879 µg/mL, suggesting the cells overexpressing ERRα have a higher tolerance to DDP (Fig. 3D). These findings indicate that siERRα increases the cytotoxicity of DDP in KLE and HEC-1A cells and promotes cell pyroptosis. Overexpression of ERRα inhibits the NLRP3/caspase-1/GSDMD pathway, helps cells resist pyroptosis, and induces resistance to DDP in EC cells.

### Upregulation of ERRα activates glycolytic metabolism and results in pyroptosis resistance in EC cells

ERRα is a transcription factor involved in the transcriptional regulation of various genes, especially those involved in metabolic signaling pathways. Following treatment with DDP, ERRα-overexpressing EC cells were imaged using TEM, which showed that the structure of the mitochondrial membrane was relatively clear, matrix was partially dissolved, and cristae were slightly broken. In contrast, in *ERRα*-knockdown EC cells, the mitochondrial membrane structure was indistinctly dissolved, the matrix was dissolved in a large area, and the crest was broken (Fig. 3E). The oxygen consumption rate (OCR) test revealed that DDP-induced mitochondrial structural destruction accompanied the impairment of basal respiration, ATP-linked respiration, maximal respiration, and spare respiratory capacities (comparison with the status in the KLE and HEC-1A groups) (*p* < 0.05) (Fig. 3F). EC cells overexpressing ERRα had a higher OCR than KLE and HEC-1A cells, whereas *ERRα*-knockdown EC cells had a lower OCR (*p* < 0.05). This finding demonstrates that the structural destruction of mitochondria corresponds to the impairment of the cell energy supply. Therefore, we further investigated whether ERRα will change the metabolic mode of EC cells and thus enhance the cells’ resistance to pyroptosis. Whether the expression of ERRα affects glycolytic metabolism of tumor cells was also determined by assessing the glycolytic ability and extracellular acidification rate (ECAR) in KLE and HEC-1A cells with different ERRα expression levels. The expression of glycolytic genes such as *GLUT1*, *PKM2*, *PDK1*, *LDHA*, and *HK2* was increased in EC cells with high ERRα expression compared with that in the controls. In contrast, the expression of all glycolytic genes decreased in *ERRα*-knockdown EC cells (*p* < 0.05) (Fig. 3G). EC cells overexpressing ERRα had a higher ECAR than HEC-1A and KLE cells, whereas *ERRα*-knockdown EC cells had a lower ECAR (Fig. 3H). Furthermore, ERRα -regulated glycolysis reprogramming was related to pyroptosis. Compared with that in the KLE^+DDP^ and HEC-1A^+DDP^ groups, the expression of pyroptosis genes was low in EC cells with high ERRα expression (*p* < 0.05). When EC cells overexpressing ERRα were pretreated with 2-deoxy-glucose (2-DG, a competitive glycolysis inhibitor), the expression of pyroptosis genes was upregulated compared with that in the KLE^−ovERRα+DDP^ and HEC-1A^−ovERRα+DDP^ groups. This finding shows that ERRα can regulate glycolysis and affect cell pyroptosis (Fig. 3I). The intrinsic relationship between glycolysis and pyroptosis has been previously illustrated. Li et al. reported that PKM2 is the key rate-limiting enzyme involved in the Warburg effect and it directly interacts with activating transcription factor 2 (ATF2) to bridge glycolysis and pyroptosis in microglia [24]. These findings demonstrate that ERRα induces pyroptosis resistance in EC cells by activating the glycolytic metabolism.

### ERRα interacts with HIF-1α to enhance pyroptosis resistance in an NLRP3-dependent manner

**Fig. 4:**
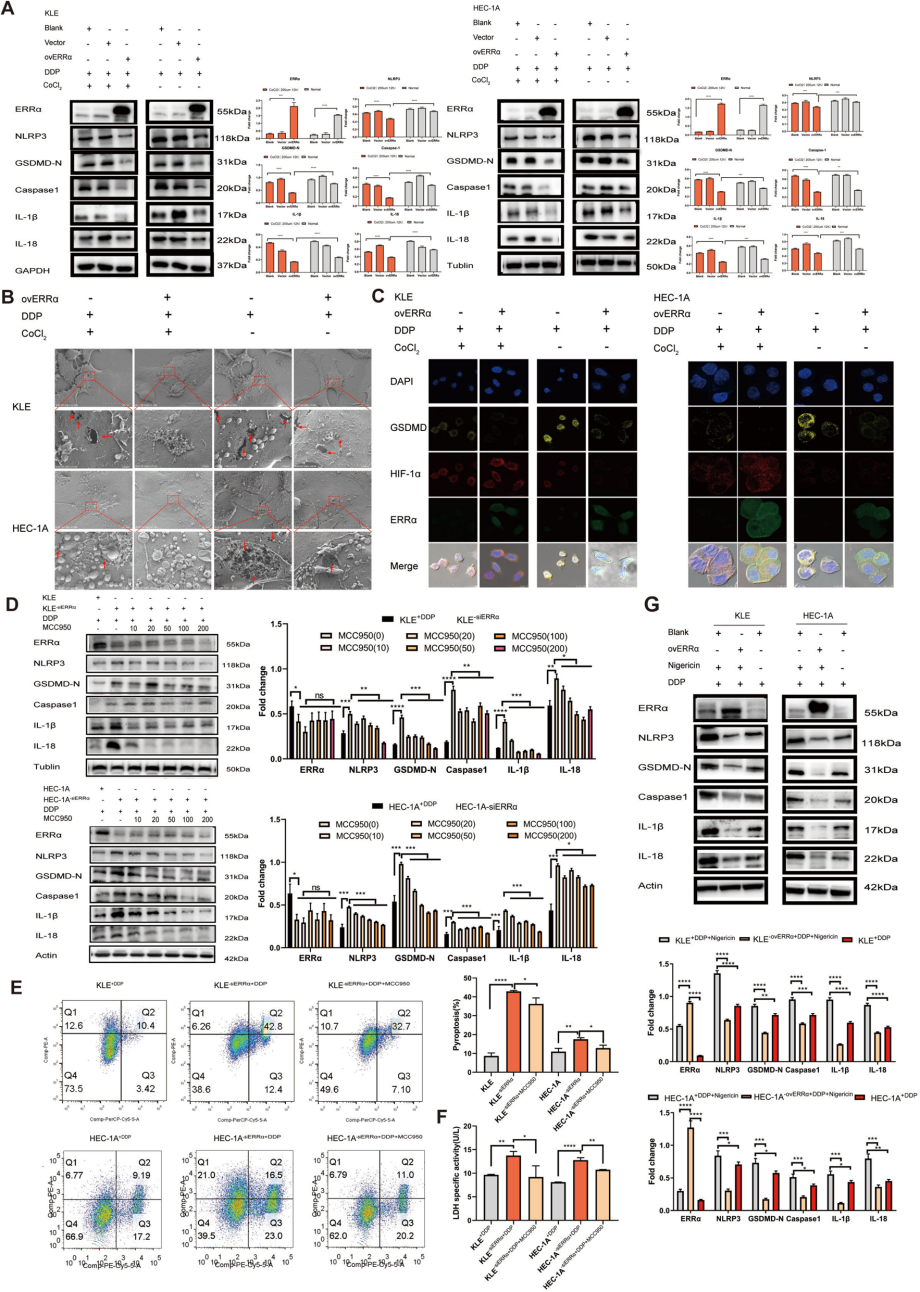
Estrogen-related receptor alpha (ERRα) interacts with HIF-1α and enhances pyroptosis resistance in an NLRP3-dependent manner. **A** Western blot (WB) analysis of pyroptosis-related proteins in KLE and HEC-1A cell lysates of the ovERRα group treated with CoCl_2_ (200 µM) for 12 h; the groups not treated with CoCl_2_ were used as controls. Each target protein was exposed on the same gel to ensure the same exposure conditions. **B** Representative SEM images of KLE and HEC-1A cells and the ovERRα group treated with CoCl_2_ (200 µM) for 12 h; the groups not treated with CoCl_2_ were used as controls. Scale: 5–30 μm. **C** Representative images of immunofluorescence staining of HIF-1α- and GSDMD-expressing cells per well, obtained using fluorescence microscopy after cisplatin (DDP) treatment of the ovERRα and control groups for 12 h. The groups not treated with CoCl_2_ were used as controls. The results represent the average of three random fields per sample. Scale: 10 µm. **D**-**F** Effect of MCC950 (10 µM) treatment on the siERRα group of KLE and HEC-1A cells measured using WB (12 h), flow-cytometry analyses (24 h), and the LDH release assay (12 h). The siERRα groups treated with DDP were included as controls. **G** Effect of nigericin (10 µM) treatment on the ovERRα group of KLE and HEC-1A cells measured using WB (12 h). KLE and HEC-1A cells treated with DDP were included as control groups. The dose of DDP in the above-mentioned in vitro experiments was 7 µg. The results represent the average of three experimental replicates. Data are shown as the mean ± SD. Statistical tests: Student’s *t*-test. *p < 0.05; **p < 0. 01; ***p < 0.001; ****p < 0.0001. Abbreviations: SEM, scanning electron microscopy

We determined whether cells overexpressing ERRα are more resistant to hypoxia and interact with HIF-1α to resist cell pyroptosis by treating KLE^−ovERRα^ and HEC-1A^−ovERRα^ cells with CoCl_2_ (200 µM) for 12 h and detecting the expression of pyroptosis-related proteins. The results showed that the expression of pyroptosis-related proteins was lower under hypoxic than under normoxic conditions. Furthermore, EC cells overexpressing ERRα under hypoxia presented lower expression of pyroptosis-related proteins than those under normal oxygen conditions (Fig. 4A). Scanning electron microscopy (Fig. 4B) revealed that the degree of cell rupture under CoCl_2_ treatment was significantly lower than that without CoCl_2_ treatment. Compared with that in the non-CoCl_2_ treatment group, the membrane of EC cells overexpressing ERRα was intact in the CoCl_2_ group, which is consistent with the immunofluorescence results. We determined the expression of the pyroptosis executive protein GSDMD via immunofluorescence and found that in the CoCl_2_ treatment group, cells overexpressing ERRα had the weakest GSDMD staining intensity. Compared with that in cells not treated with CoCl_2_, the expression of GSDMD in the CoCl_2_-treated group decreased (Fig. 4C). The expression of HIF-1α increases in the hypoxic tumor microenvironment, and the interaction of HIF-1α with EC cells overexpressing ERRα can enhance the resistance of tumor cells to pyroptosis. Additionally, to illustrate that the regulation of EC cell pyroptosis by ERRα depends on NLRP3, we used MCC950, a specific NLRP3 inhibitor, in EC cells treated with siERRα. As shown in Fig. 4D, the levels of pyroptosis-related proteins were significantly decreased in a dose-dependent manner with increasing MCC950 concentration. Flow cytometry and the LDH release assay showed (Fig. 4E-F) that DDP increased the pyroptotic rate and LDH release of cells with low ERRα expression, but both decreased significantly upon pretreatment with MCC950. We also used nigericin, a K^+^ ionophore that activates the NLRP3 inflammasomes [25], to treat EC cells overexpressing ERRα. It was found that nigericin could upregulate pyroptosis-related protein expression. Further, the degree of pyroptosis was also reduced in the ERRα-overexpression group (*p* < 0.05) (Fig. 4G). These results suggest that the regulation of EC cell pyroptosis by ERRα depends on NLRP3, and cells become more resistant to pyroptosis through interaction with HIF-1α.

### Downregulation of ERRα expression increases the sensitivity of EC organoids to DDP

**Fig. 5:**
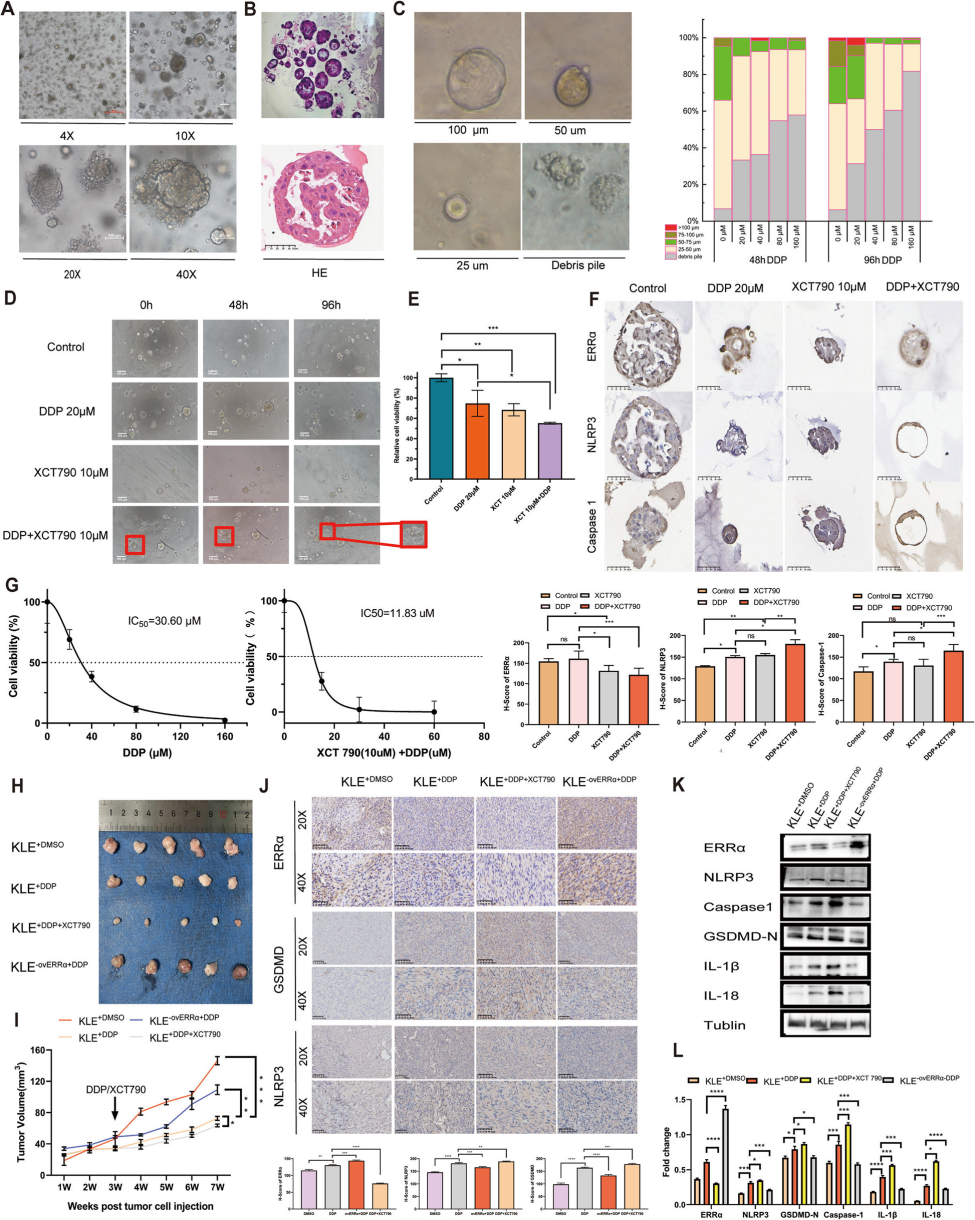
Knockdown of *ERRα* inhibits tumor growth and promotes DDP sensitivity in EC organoids and a xenograft animal model. **A** Cell morphology of organoids at different magnifications was recorded using an optical microscope. Scale: 50, 100, and 500 µm. **B** The histological structure and morphology of EC organoids were verified using H&E staining. **C** Fraction of organoids of different sizes after treatment with DDP at different concentrations is shown. The amount of organoid debris following treatment with DDP at different concentrations increased, and the shape and size of the debris were recorded under a microscope. **D** Inhibitory effect of DDP (20 µM) and XCT790 (10 µM) alone or in combination with EC organoids was recorded using an optical microscope. The group treated with DMSO was used as the control group. Scale: 100 µm. **E** Evaluation of cell viability following treatment with DDP (20 µM) and XCT790 (10 µM) alone or in combination in each experimental group using the CellTiter-Glo assay. Cells treated with DMSO were used as the control group. **F** Representative images of the IHC of ERRα, NLRP3, and caspase-1 in EC organoids are shown (the values in each graph represent the average of three random fields per sample). The groups treated with DMSO and DDP alone were used as the control groups. **G** After pretreatment with or without XCT790 (10 µM), the IC_50_ of DDP in EC organoids was determined using the CCK8 assay. **H** Representative images of tumors captured at the end of the study and tumor size in BALB/c nude mice of the four treatment groups. **I** Tumor growth curves of the four treatment groups are shown. The KLE^+DMSO^ and KLE^+DDP^ treatment groups were used as control groups (day 0 was defined as the day when different cell groups were implanted). **J** Representative images of the IHC of ERRα, NLRP3, and GSDMD in tumor sections from xenograft mice are shown (each point represents the average of three random fields per sample). The KLE^+DMSO^ and KLE^+DDP^ treatment groups were used as control groups. **K**-**L** WB analysis of ERRα, NLRP3, caspase-1, GSDMD-N, IL-18, and IL-1β in tumor specimens of xenograft mice. Tubulin was used as a loading control. The KLE^+DMSO^ and KLE^+DDP^ treatment groups were used as control groups. Data are shown as the mean ± SD. Statistical tests: Student’s *t*-test. *p < 0.05; **p < 0.01; ***p < 0.001; ****p < 0.0001. Abbreviations: H&E, hematoxylin and eosin

To better simulate the in vivo conditions, a three-dimensional organ-like model was used to establish EC-derived organoids and the sensitivity of these organoids with different ERRα expression levels to DDP was analyzed. The cell morphology of organoids at different magnifications was recorded using an optical microscope (Fig. 5A). The histological structure and cell morphology of EC organoids were examined using hematoxylin and eosin staining (Fig. 5B). EC organoids treated with DDP at different gradient concentrations were observed under a microscope, and the number, shape, and size of the organoids were recorded (Fig. 5C). The fraction of debris of organoids after DDP treatment increased significantly in a dose-dependent manner. Next, we assessed the effects of DDP and XCT790 (a specific ERRα inhibitor) on the organoids based on the drugs alone or in combination to test whether XCT790 enhanced the sensitivity of EC to DDP. Different treatment groups were established (Fig. 5D): DMSO, DDP (20 µM), XCT790 (10 µM), and DDP + XCT790. The results showed no obvious morphological changes between the single-drug experimental group and untreated control group. However, obvious morphological changes (shrinkage, apoptosis, and hollow vesicles) were observed in the combined experimental group, and the degree of change became more evident in the DDP + XCT790 group. The cell survival rates of each experimental group were as follows (Fig. 5E): DDP 20 μM (74.76%), XCT790 10 μM (68.37%), and DDP + XCT790 10 μM (55.18%). The effect of DDP combined with XCT790 at 10 μM on cell viability was more significant than that in the other groups. We then verified the effect of different ERRα expression levels on cell pyroptosis and cell sensitivity to DDP. As shown in Fig. 5F, the expression of pyroptosis-related proteins (NLRP3 and caspase-1) was higher in the DDP group than in the DMSO control group. However, their expression was more prominent in the DDP + XCT790 group. The IC_50_ was approximately 30.60 μM for EC organoids and 11.83 μM for EC organoids pretreated with XCT790 (Fig. 5G). This finding confirmed that the inhibition of ERRα promotes the activation of the NLRP3/caspase1/GSDMD pyroptosis pathway in EC cells, which may induce sensitivity to DDP in the EC organoid model.

### ERRα downregulation inhibits tumor growth and promotes DDP sensitivity in a xenograft animal model

To determine whether ERRα inhibits pyroptosis through the NLRP3/caspase-1/GSDMD pathway, enhances resistance to DDP in vivo, and promotes the growth of EC xenografts, we established a model for the xenotransplantation of KLE cells in nude mice. Stable *ERRα*-overexpressing cells and KLE cells were transplanted into the armpits of immunodeficient BALB/c nude mice to generate the xenograft mouse model. Three weeks after tumor cell transplantation, four treatment groups of xenograft mice were established. The representative images of the tumors taken at the end of the study, along with the tumor sizes in the *BALB/c* nude mice for the four treatment groups, are shown in Fig. 5H. Compared with the KLE^+DMSO^ control group, DDP treatment significantly inhibited the growth of tumor cells, and its effect was most prominent in the KLE^+DDP+XCT790^ treatment group. Tumor growth in the KLE^−ovERRα+DDP^ group was significantly faster than that in the KLE^+DDP^ group (Fig. 5I), indicating that ERRα overexpression promotes tumor growth and inhibits DDP sensitivity in vivo.

Next, we used immunohistochemical staining (IHC) to verify whether ERRα overexpression affects the NLRP3/caspase-1/GSDMD pathway in vivo. As shown in Fig. 5J, the expression of pyroptosis-related proteins (NLRP3 and GSDMD) was higher in the DDP group than in the DMSO group. However, their expression was more prominent in the KLE^+DDP+XCT790^ group and not very prominent in the KLE^−ovERRα+DDP^ group. Compared with that in the KLE^−ovERRα+DDP^ group, DDP induced the upregulation of NLRP3, caspase-1, GSDMD-N, IL-18, and IL-1β in the KLE^+DDP^ group. Importantly, the expression of these pyroptosis-related proteins was considerably high in the KLE^+DDP+XCT790^ group (Fig. 5K-L). Furthermore, ERRα overexpression inhibited the NLRP3/caspase-1/GSDMD pathway activation and cell pyroptosis in vivo. No significant hepatotoxicity or weight loss was observed in the DDP or DDP + XCT790 treatment groups (data not shown). In summary, our findings show that ERRα inhibits pyroptosis through the NLRP3/caspase-1/GSDMD pathway and enhances resistance to DPP in EC cells.

### ERRα is involved in cell pyroptosis and participates in the reprogramming of energy metabolism based on an analysis of The Cancer Genome Atlas data

**Fig. 6:**
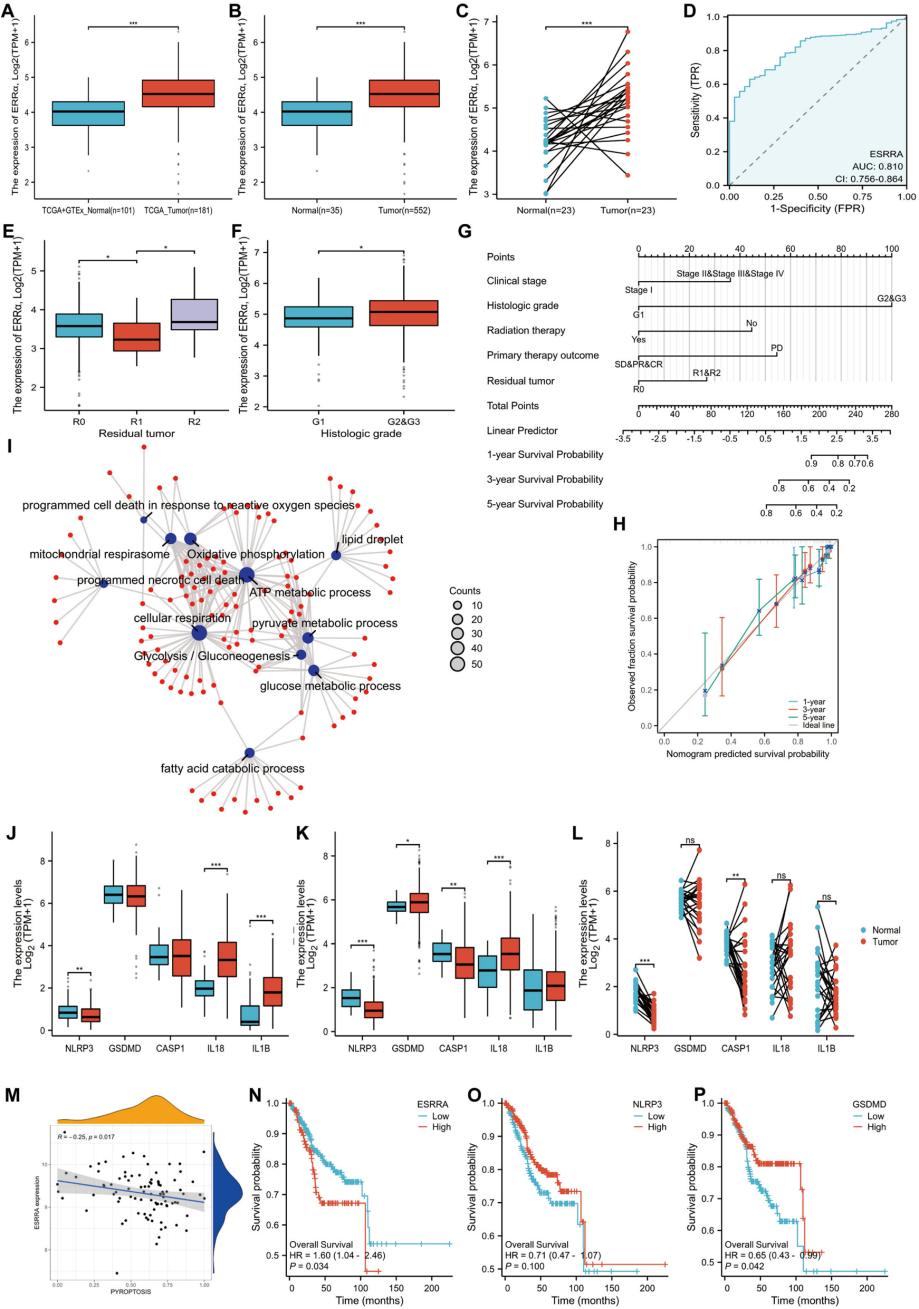
Estrogen-related receptor alpha (ERRα) is involved in cell pyroptosis and participates in the reprogramming of energy metabolism based on TCGA data analysis. **A** Differential expression map of ERRα. ERRα expression in GTEx (78 cases) combined with TCGA paracancerous (23 cases) and TCGA endometrial cancer (181 cases) samples. Wilcoxon rank sum test was used for statistical analysis. **B** ERRα expression in normal (35 cases) and endometrial cancer (EC) tissues (552 cases) from TCGA. Wilcoxon rank sum test was used for statistical analysis. **C** ERRα expression in EC cancer tissues (23 cases) and the corresponding normal tissues from TCGA database. Paired sample *t-*test was used for statistical analysis. **D** ROC curves shows that ERRα (known as ESRRA) has high diagnostic accuracy in EC. **E**–**F** Associations between ERRα expression and residual tumor and histologic grade. **G** Using the Cox proportional hazard regression model, clinical stage, histologic grade, radiation therapy, primary treatment outcome, and residual tumor were included in the nomogram for predicting the probability of 1-, 3-, and 5-year OS in patients with EC. **H** Calibration plot of the nomogram for predicting the probability of OS in patients with EC at 1, 3, and 5 years. **I** GO analysis of TCGA data showed that ERRα-related genes in EC were enriched in glucose metabolism, pyruvate metabolism, programmed necrotic cell death, and glycolysis/gluconeogenesis. The subset of representative terms from this cluster was visualized with the *p*-value set to 0.05. **J**) Differential expression map of pyroptosis-related genes. The expression of pyroptosis-related genes in GTEx (78 cases) combined with TCGA paracancerous (23 cases) and TCGA endometrial cancer (181 cases) samples. Wilcoxon rank sum test was used for statistical analysis. **K** Pyroptosis-related gene expression in normal (35 cases) and EC tissues (552 cases) from TCGA. Wilcoxon rank sum test was used for statistical analysis. **L** Expression of pyroptosis-related genes in EC tissues (23 cases) and the corresponding normal tissues in TCGA database. Paired sample *t*-test was used for statistical analysis. **M** Analysis of GEO database data to show the correlation between the expression of ERRα and the pyroptosis pathway. **N**-**P** Survival analysis using ERRα,NLRP3, and GSDMD expression in EC. OS of patients with high versus low ERRα, NLRP3, and GSDMD expression levels. *p < 0.05; **p < 0.01; ***p < 0.001. Abbreviations: ERRα, estrogen-related receptor alpha; TCGA, The Cancer Genome Atlas; ROC, receiver operating characteristic; GO, Gene Ontology; OS, overall survival; ns, no statistical significance

We previously demonstrated that ERRα is highly expressed in patients with EC compared with that in paracancerous tissues, and is involved in EC progression [16]. We used bioinformatics to analyze the expression of ERRα in normal tissues in the GTEx database (78 cases) and in paracancerous tissues (23 cases) and EC samples (181 cases) from The Cancer Genome Atlas (TCGA) database. The expression of ERRα in normal tissues (35 cases) and EC tissues (552 cases), based on TCGA data was analyzed. Simultaneously, the expression of ERRα in EC samples (23 cases) from TCGA data and their adjacent tissues was also compared. The results showed that ERRα was highly expressed in EC samples (*p* < 0.001) (Fig. 6A–C). In addition, the area under the receiver operating characteristic (ROC) curve was 0.810 (95% CI: 0.756–0.864) (Fig. 6D), which indicates that ERRα (also known as ESRRA) has high diagnostic accuracy in EC. As shown in Supplement Table 3, 552 primary EC cases with clinical information and gene expression data were retrieved from TCGA database. The average age of the 552 patients was 64 years. Correlation analysis showed that *ERRα* expression correlates with race (*p* = 0.003), histologic grade (*p* = 0.002), radiation therapy (*p* = 0.030), and residual tumor (*p* = 0.010). No correlation was observed between ERRα expression and other clinicopathological features. The details are presented in Fig. 6E–F. Age (*p* = 0.009), histological type (*p* < 0.001), clinical stage (*p* < 0.001), histologic grade (*p* < 0.001), tumor invasion (*p* < 0.001), radiation therapy (*p* = 0.019), primary therapy outcome (*p* < 0.001), and residual tumor (*p* < 0.001) were included in a Cox proportional risk regression model for multivariate analysis. As shown in Supplement Table 4, multivariate analysis showed that clinical stage, histologic grade, radiation therapy, primary treatment outcome, and residual tumor are independent risk factors for the overall survival (OS) in EC patients (*p* < 0.05). Based on analysis using the Cox proportional hazards regression model, clinical stage, histologic grade, radiation therapy, primary treatment outcome, and residual tumor were included in the nomogram (Fig. 6G). The concordance index of the prognostic model was 0.815 (95% CI: 0.787–0.842). We constructed a calibration chart to evaluate the consistency between the predicted and actual overall survival. The prediction results of the nomogram were reliable (Fig. 6H).

Gene Ontology (GO) analysis was performed to provide a better understanding of the potential biological functions of ERRα in EC and identify the ERRα-related genes in EC from TCGA database. The enriched biological processes were ATP metabolic process, cellular respiration, glucose metabolic process, pyruvate metabolic process, fatty acid catabolic process, programmed necrotic cell death, and programmed cell death in response to reactive oxygen species. The cellular components group included mitochondrial respirasome and lipid droplets. The enriched Kyoto Encyclopedia of Genes and Genomes (KEGG) pathways included oxidative phosphorylation and glycolysis/gluconeogenesis (Fig. 6I).

The above analysis suggests the involvement of ERRα in EC energy metabolism. To analyze the effect of ERRα on cell pyroptosis, we first analyzed the expression of pyroptosis-related genes in GTEx combined with that in TCGA paracancerous tissues and TCGA EC samples (Fig. 6J–L). IL-18 and IL-1β were highly expressed in EC samples (*p* < 0.001), whereas the expression of NLRP3 in tumors was lower than that in normal tissues (*p* = 0.001). There was no significant difference in the expression of *GSDMD* and *CASP1* (Caspase 1) between the tumor and normal tissues. The expression of pyroptosis-related genes was compared in 35 paracancerous tissues from TCGA and 552 EC tissues. GSDMD (*p* = 0.012) and IL-18 (*p* < 0.001) were highly expressed in tumor tissues, whereas *NLRP3* (*p* < 0.001) and *CASP1* (*p* = 0.001) expression was low in tumor tissues. IL-1β expression in tumors was higher than that in paracancerous tissues, but the difference was not significant (*p* > 0.05). A comparison of the expression of the pyroptosis-related genes in 23 EC samples from TCGA and their adjacent tissues revealed that the expression of *NLRP3* (t =  − 9.641, *p* < 0.001) and *CASP1* (t =  − 3.215, *p* = 0.004) in tumors was lower than that in paracancerous tissues. There was no significant difference in the expression of GSDMD, IL-18, and IL-1β between EC samples and their matched adjacent tissues. Moreover, we collected information for subsequent analyses of GEO data, in which dataset GSE17025 contained EC tissues (91 cases) and normal tissues (12 cases). The results showed that there was a significant negative correlation between the expression of ERRα and pyroptosis pathway genes (Fig. 6M).

To determine the correlation of the expression of ERRα and pyroptosis-related genes with the prognosis of EC, we compared the survival rates of patients overexpressing and underexpressing ERRα. The Kaplan–Meier survival analysis revealed that patients with EC in the ERRα-overexpression group had a poor overall survival (OS) (HR = 1.60, 1.04–2.46, *p* = 0.034) (Fig. 6N), and the OS was higher in patients with EC overexpressing GSDMD (HR = 0.65, 0.43–0.99, *p* = 0.042). Whereas the OS was not significant difference between NLRP3 low/high expression group, but there was a tendency of the lower the expression of NLRP3, the worse of the OS (Fig. 6O-P). The above results from TCGA database indicate that high ERRα expression is associated with cell pyroptosis and is involved in the reprogramming of energy metabolism.

## Discussion

The initiation of metabolic reprogramming and regulation of pyroptosis in tumor cells are emerging research topics of interest. As a central regulator of cellular energy metabolism, ERRα participates in the vital processes of the tricarboxylic acid cycle and energy, glucose, and lipid metabolism [26]. High ERRα expression corresponds to a more aggressive and malignant tumor phenotypes, such as the phenotypes associated with chemotherapy resistance and tumor metastasis, which are closely related to poor clinical prognosis [21]. Inhibition of ERRα expression can block mitochondrial respiration and enhance the anti-cancer effects of chemotherapy [27]. However, data regarding the nuclear transcriptional regulator ERRα and tumor cellular pyroptosis remain limited. In this study, to the best of our knowledge, we demonstrated, for the first time, that the upregulation of ERRα leads to glycolytic metabolic reprogramming and induces resistance to pyroptosis by targeting NLRP3, which results in DDP resistance in EC cells in vivo and in vitro. Moreover, this effect could be triggered and enhanced in the hypoxic tumor microenvironment.

Growth adaptation in hypoxic microenvironment is a common characteristic of solid tumors [28]. Zou et al. showed that ERRα could physically interact with HIF-1α through its AF-2 domain and promote the growth of tumor cells involved in angiogenesis and glycogenesis [29], while Ao et al. reported that these ERR family members only interact with functional HIF-1 heterodimers [30]. In addition, this ERR-HIF-1 interaction requires the complete DNA-binding domain of ERR. The results of these studies are somewhat different; this may be due to the different experimental methods adopted in each of these studies. In our study, we screened the interaction between HIF-1α and ERRα using the STRING database and verified the results by performing co-immunoprecipitation analyses using EC cells. Furthermore, we revealed that the crosstalk between HIF-1α and ERRα was significantly consolidated in EC cells under a hypoxic microenvironment, simulated by CoCl_2_ (a classic hypoxic agonist). One of the major mechanisms whereby tumor cells mediate hypoxic responses involves the upregulation of HIF-1α, which affects metabolic reprogramming [31]. After treatment with CoCl_2_, the upregulation of HIF-1α in EC cells promotes ERRα overexpression. In the present study, EC cells with high ERRα expression were found to be more resistant to pyroptosis.

Recently, pyroptosis has been studied in the treatment of malignant tumors. In 2017, Wang et al. reported that cisplatin, topotecan, and etoposide could promote tumor cell pyroptosis [32]. Tanshinone IIA can promote pyroptosis in cervical cancer HeLa cells and exert anticancer activity by regulating the mir-145/GSDMD signaling [33]. Hydrogen inhibits EC growth via the NLRP3/caspase-1/GSDMD-mediated pyroptosis pathway by promoting ROS accumulation [34]. Interestingly, the overexpression of ERRα inhibited the expression of apoptosis-related proteins such as NLRP3, whereas the knockdown of *ERRα* significantly promoted pyroptosis in EC cells and increased their sensitivity to cisplatin. We predicted the binding site of ERRα in the -800 to -400 promoter region of *NLRP3* using the JASPAR database. Furthermore, the dual-luciferase reporter assay revealed that ERRα initiates the transcription of *NLRP3* by directly binding to the sequence 3′-ACAACTTGAACACGGAAACG-5′. To determine whether this regulation of pyroptosis by ERRα in EC cells is dependent of NLRP3-mediated signaling, we treated EC cells underexpressing ERRα with MCC950, a specific NLRP3 antagonist. With the increase in MCC950 concentration, the expression of pyroptosis-related proteins decreased in a gradient, highlighting a close correlation between ERRα and pyroptosis. Finally, the effects of ERRα on the sensitivity of EC to DDP and resistance to pyroptosis were further verified in EC-derived organoids as well as in nude mice. Organ-like models provide a preclinical platform for the individualized treatment of patients with malignant cancer [35–39]. The results showed that the overexpression of ERRα promoted the growth of EC tumors in nude mice and inhibited the activation of the NLRP3/caspase-1/GSDMD pathway. After treatment with XCT790, a specific antagonist of ERRα, tumor growth inhibition and increased sensitivity to DDP were observed. Data from TCGA database showed that the overexpression of the pyroptosis executive gene GSDMD is positively correlated with the overall survival of EC, which indicated that pyroptosis is related to the prognosis of EC. Therefore, our findings indicate that ERRα plays a key role in the response to cisplatin chemotherapy by regulating pyroptosis.

The cholesterol and mevalonate pathways are involved in the progression, invasion, and drug resistance of breast cancer by activating the ERRα pathway [40]. Previously, we established that the TFEB-ERRα axis induces lipid reprogramming and promotes pseudopodium formation in EC cells, enhancing cell membrane fluidity and promoting the invasion and metastasis of EC [16]. However, the mechanism through which ERRα induces resistance to chemotherapy requires further exploration. Tumor cells show an enhanced glycolysis ability to meet their high energy demand. Moreover, the aerobic glycolysis rate is related to the occurrence, progression, and drug resistance of cancers [41]. Zeng et al. reported that miR-211-5p mediates carcinogenic effects by inhibiting pyroptosis and enhancing glycolysis in low-metastatic melanoma tumor cells by regulating the expression of the target gene guanine nucleotide-binding protein subunit α-15 (*GNA15*) [42]. ERRα is a key regulator of energy metabolism, and TCGA dataset analysis showed that ERRα-related genes are involved in glucose metabolism and programmed cell death. These results indicate that the overexpression of ERRα could promote glycolysis, increase the ECAR in EC cells, and enhance their resistance to pyroptosis. This may represent the main crosstalk between the glycolytic reprogramming and pyroptosis of EC cells induced by chemotherapy. Compared with EC cells with ERRα knockdown, cells overexpressing ERRα also showed a high IC_50_ for DDP. It was confirmed that the high expression of ERRα leads to DDP resistance through pyroptosis inhibition and the glycolytic reprogramming of EC cells. Daniela et al. found that C13, a drug-resistant ovarian cancer cell line, was more sensitive to glucose deprivation, and the drug-resistant cells were more dependent on glucose to maintain their phenotype [43]. A recent study showed that glycolytic metabolism was upregulated in hepatocellular carcinoma cells and that this is related to sorafenib resistance [44]. He et al. discussed the potential role of metabolic reprogramming in acquired drug resistance of osteosarcoma and the regulatory mechanisms underlying this process; these authors showed that increased ERRα expression is related to the metabolic reprogramming of chemotherapy-resistant osteosarcoma cells, because the targeted inhibition of ERRα expression restores the transformation of the metabolic mode [45]. Based on our results and previous findings, we conclude that ERRα overexpression could lead to drug resistance in EC by enhancing pyroptosis resistance and the expression of glycolysis-related genes in tumor cells.

## Conclusions

**Fig. 7:**
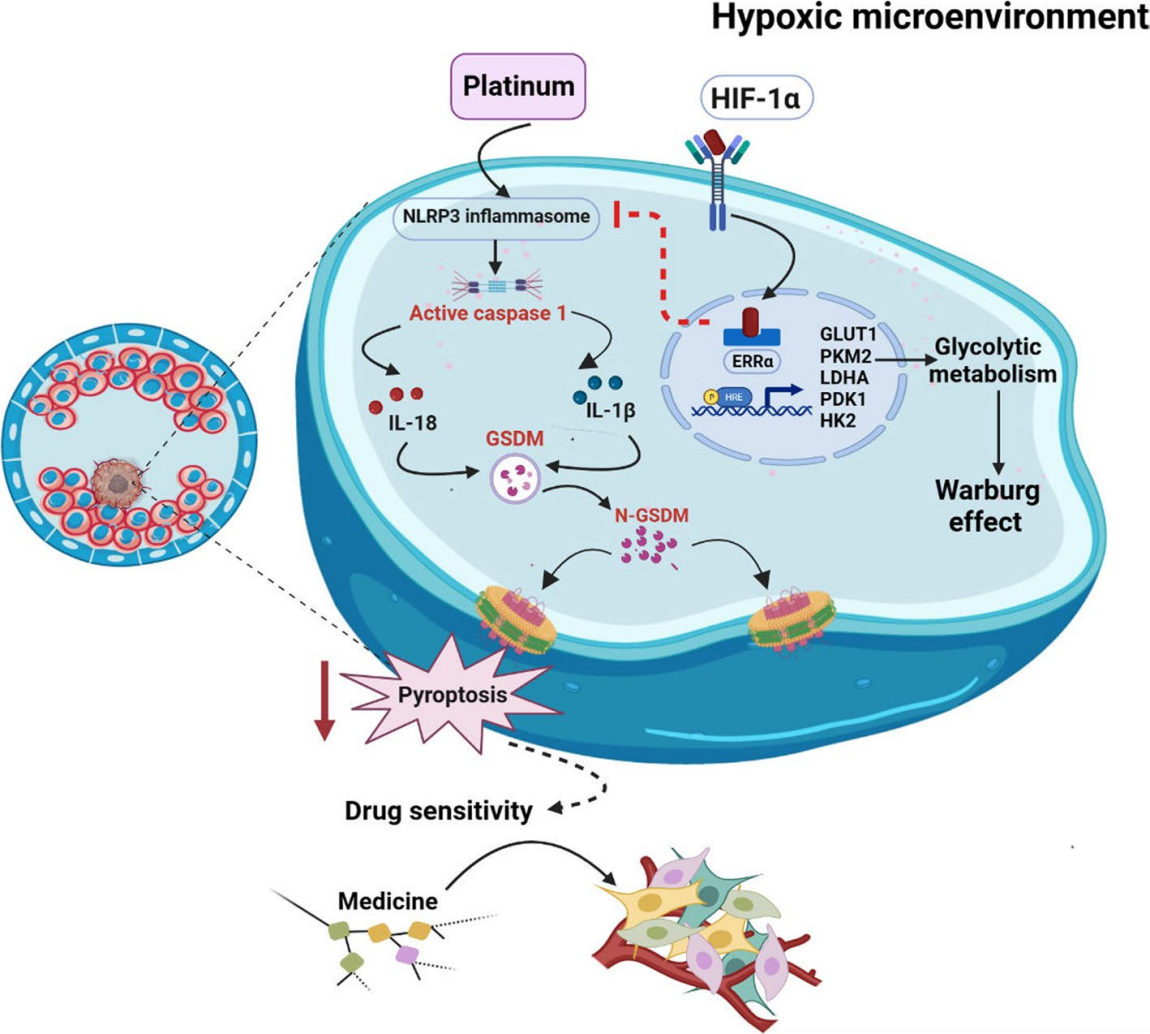
Molecular mechanisms underlying the regulation of estrogen-related receptor alpha (ERRα) expression in pyroptosis. ERRα promotes the resistance of EC cells to pyroptosis by regulating NLRP3 inflammasomes. Platinum activates the inflammasome components, followed by the cleavage of CASP1 (Caspase 1) and production of inflammatory factors such as IL-1β and IL-18, and then triggers the cleavage of GSDMD and promotes cell membrane rupture by forming GSDMD-N. In addition, HIF-1α interacts with ERRα and enhances glycolytic metabolism in cancer cells to resist chemotherapy. Moreover, this effect might be triggered and enhanced by the hypoxic tumor microenvironment

The crosstalk between HIF-1α and ERRα was consolidated. High expression of ERRα, triggered by the hypoxic microenvironment, enhances cell resistance to pyroptosis by direct target binding to the promoter of NLRP3 with the sequence 3′-ACAACTTGAACACGGAAACG-5′, inhibiting the downstream pyroptosis signaling pathway. Moreover, overexpression of ERRα participates in the malignant progression of EC through the reprogramming of glycolysis, accompanied by increased extracellular acidification rate, which leads to the resistance of EC cells to pyroptosis and cisplatin chemotherapy (Fig. 7). These findings highlight that the mechanism underlying the role of ERRα in chemotherapeutic resistance involves regulating the cell pyroptosis signaling pathway, which is a potential novel clinical treatment target.

## Supplementary Information

**Additional file 1.** 

**Additional file 2.** 

**Additional file 3.** 

**Additional file 4.** 

**Additional file 5.** 

**Additional file 6.** 

**Additional file 7.** 

**Additional file 8.** 

**Additional file 9.**

## Data Availability

The datasets used and/or analysed during the current study are available from the corresponding author on reasonable request.

## Abbreviations

EC: Endometrial cancer
ERRα: Estrogen-related receptor alpha
HIF-1α: Hypoxia-inducible factor-1 alpha
IC50: 50% Inhibitory concentration
CCK8: Cell Counting Kit-8
PI: Propidium iodide
CUT&Tag: Cleavage under targets and tagmentation
LDH: Lactate dehydrogenase
TEM: Transmission electron microscopy
WB: Western blot
UCSC: University of California Santa Cruz
OCR: Oxygen consumption rate
ECAR: Extracellular acidification rate
HE: Hematoxylin and eosin
IHC: Immunohistochemical staining
TCGA: The Cancer Genome Atlas
GEO: Gene Expression Omnibus
ROC: Receiver operating characteristic
OS: Overall survival
GO: Gene Ontology
KEGG: Kyoto Encyclopedia of Genes and Genomes
HR: Hazard ratio
CI: Confidence interval
