# Correction: ERRα promotes glycolytic metabolism and targets the NLRP3/caspase-1/GSDMD pathway to regulate pyroptosis in endometrial cancer

**DOI:** 10.1186/s13046-025-03292-z

**Published:** 2025-01-25

**Authors:** Pingping Su, Xiaodan Mao, Jincheng Ma, Lixiang Huang, Lirui Yu, Shuting Tang, Mingzhi Zhuang, Zhonglei Lu, Kelvin Stefan Osafo, Yuan Ren, Xinrui Wang, Xite Lin, Leyi Huang, Xiaoli Huang, Elena Ioana Braicu, Jalid Sehouli, Pengming Sun

**Affiliations:** 1 Laboratory of Gynecologic Oncology, Department of Gynecology, College of Clinical Medicine for Obstetrics & Gynecology and Pediatrics, Fujian Maternity and Child Health Hospital, Fujian Medical University, Fuzhou, 350001 Fujian China; 2 Women and Children’s Hospital, School of Medicine, Xiamen University, Xiamen, China; 3 Fujian Key Laboratory of Women and Children’s Critical Diseases Research, Fujian Maternity and Child Health Hospital, Fuzhou, 350001 Fujian China; 4 Fujian Clinical Research Center for Gynecologic Oncology, Fujian Maternity and Child Health Hospital, Fujian Obstetrics and Gynecology Hospital, Fuzhou, 350001 Fujian China; 5 College of Biological Science and Engineering, Fuzhou University, Fuzhou, 350108 Fujian China; 6 Medical Research Center, College of Clinical Medicine for Obstetrics and Gynecology and Pediatrics, Fujian Maternity and Child Health Hospital, Fujian Medical University, Fuzhou, 350001 Fujian China; 7 NHC Key Laboratory of Technical Evaluation of Fertility Regulation for Non-Human Primate, Fujian Maternity and Child Health Hospital, Fuzhou, 350001 Fujian China; 8 Department of Obstetrics and Gynecology, The First Afliated Hospital, Fujian Medical University, Fuzhou, 350005 Fujian China; 9 Department of Gynecology and Obstetrics, Charité Virchow University Hospital, Augustenberger Platz1, 13353 Berlin, Germany; 10 National Key Clinical Specialty Construction Program of China (Gynecology), Fujian Maternity and Child Health Hospital, Fuzhou, 350001 Fujian China

**Correction** **:** **J Exp Clin Cancer Res 42, 274 (2023)**

**https://doi.org/10.1186/s13046-023-02834-7**

Following the publication of the original article [1], the authors identified an error in Figure 3B where the image is identical with Figure 4E due to the misplacement of the image during submission process. The flow cytometry plot of Fig. 3B (the KLE+DDP group) needs to be replaced and corrected with the correct image.

The correct figure is presented below:

**Incorrect **Fig. 3

**Fig. 3:**
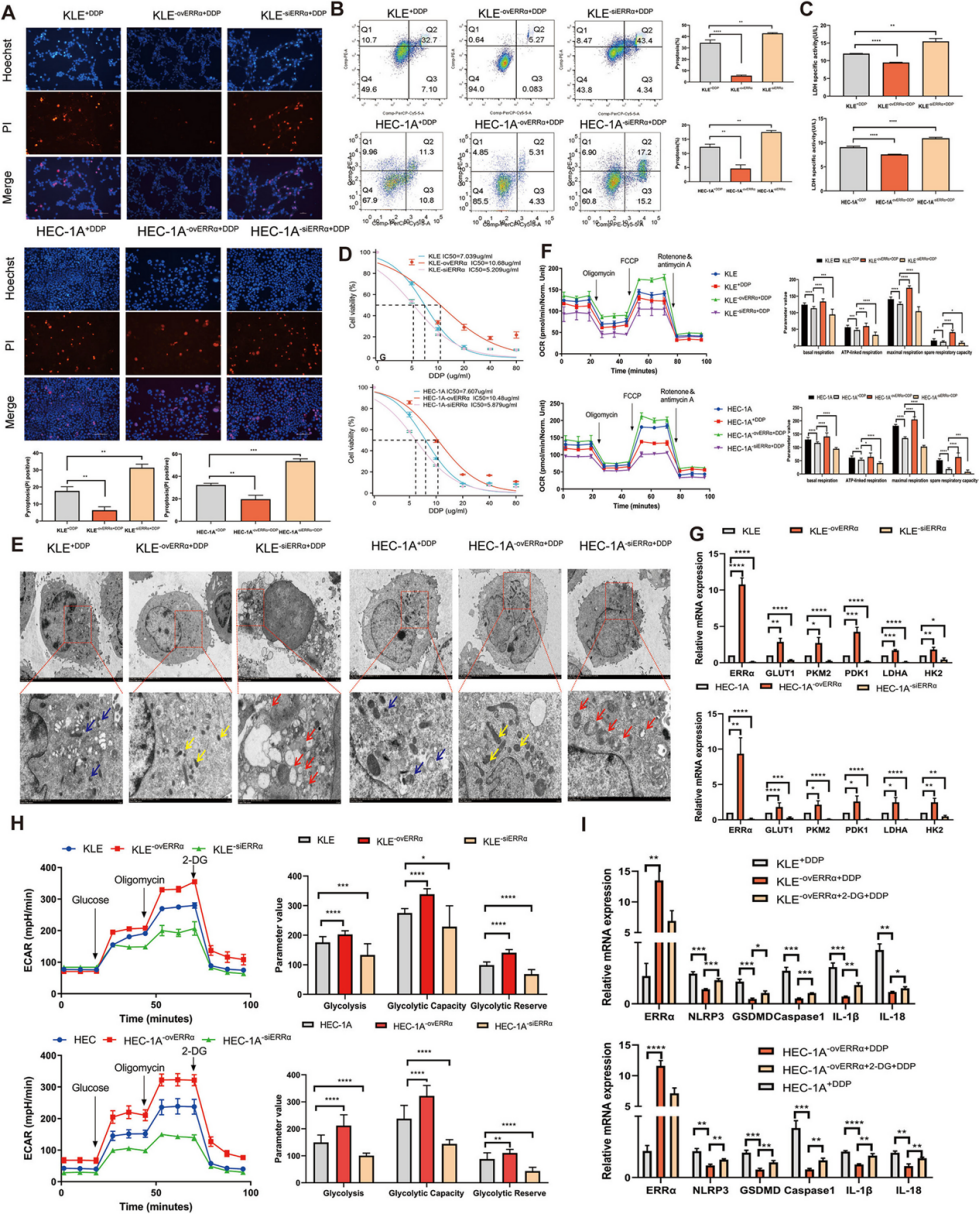
Overexpression of estrogen-related receptor alpha (ERRα) enhances pyroptosis resistance accompanied by glycolytic metabolism, leading to cisplatin (DDP) resistance of EC cells. **A** Pyroptotic cells (PI-positive) in each well were imaged using fuorescence microscopy after DDP treatment for 12 h in the ovERRα and siERRα groups. KLE and HEC-1A cells treated with DDP for 12 h were included as the control group in the experiment. The values in each graph represent the average of three random felds per sample. Scale: 100 µm. **B** Percentage of AnnexinV-PE- and 7AAD-positive pyroptotic cells within the second quadrant (Q2) from diferent ERRα expression groups was analyzed using fow cytometry after 24 h of DDP treatment. KLE and HEC-1A cells treated with DDP for 24 h were used as the control group. **C** LDH activity of cell culture supernatants was measured in diferent ERRα expression groups after DDP treatment for 12 h. KLE and HEC-1A cells treated with DDP for 12 h were included as the control group in the experiment. **D** KLE and HEC-1A cells were treated with DDP at various concentration gradients for 48 h, and the IC_50_ of DDP in diferent ERRα expression groups was determined using the CCK8 assay. **E** Representative TEM images of KLE and HEC-1A cells in diferent ERRα expression groups treated with 7 µg DDP for 12 h. The mitochondrial membrane structure was indistinctly dissolved, the matrix was dissolved in a large area, and the crest was broken in siERRα cells, as indicated with the red arrows; the yellow arrows indicate ERRα-overexpressing EC cells whose mitochondrial membrane structure was relatively clear, the matrix was partially dissolved, and the cristae were slightly broken. The blue arrows indicate the mitochondria in the KLE and HEC-1A cells of control group treated with DDP for 12 h. Scale: 2 μm. **F** Basal, ATP-linked, and maximal respiration and spare respiratory capacities were assessed to evaluate the mitochondrial function in diferent ERRα expression groups. KLE^+DDP^ and HEC-1A^+DDP^ cells were used as the control groups. **G** Total RNA in KLE and HEC-1A cells was assessed using RT-qPCR to measure the expression of glycolysis-related genes in the ovERRα and siERRα groups. KLE and HEC-1A cells were used as the control group. H Extracellular acidifcation rate, glycolysis, glycolytic capacity, and glycolytic reserve in diferent ERRα expression groups are shown. KLE and HEC-1A cells were used as the control group. I Total RNA in KLE and HEC-1A cells was assessed using RT-qPCR to measure the expression of pyroptosis-related genes in EC cells. KLE^−ovERRα+DDP^ and HEC-1A^−ovERRα+DDP^ cells were used as the control groups. The results are presented as the average of three experimental replicates. Data are shown as the mean±SD. Statistical tests: Student’s *t*-test. *p<0.05; **p<0.01; ***p<0.001; ****p<0.0001. Abbreviations: EC, endometrial cancer; IC_50_, inhibitory concentration; PI, propidium iodide; TEM, transmission electron microscopy; 2-DG, 2-deoxy-glucose

**Correct **Fig. 3

**Fig. 3:**
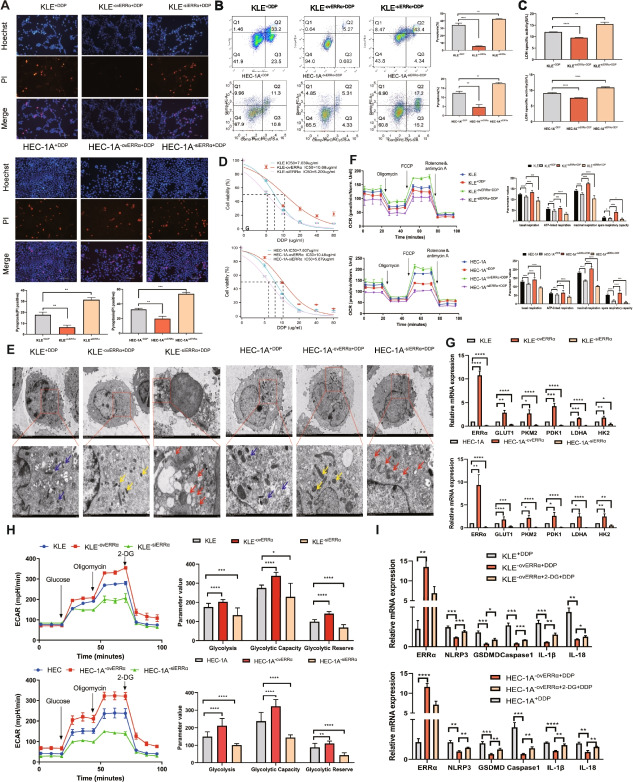
Overexpression of estrogen-related receptor alpha (ERRα) enhances pyroptosis resistance accompanied by glycolytic metabolism, leading to cisplatin (DDP) resistance of EC cells. **A** Pyroptotic cells (PI-positive) in each well were imaged using fuorescence microscopy after DDP treatment for 12 h in the ovERRα and siERRα groups. KLE and HEC-1A cells treated with DDP for 12 h were included as the control group in the experiment. The values in each graph represent the average of three random felds per sample. Scale: 100 µm. **B** Percentage of AnnexinV-PE- and 7AAD-positive pyroptotic cells within the second quadrant (Q2) from diferent ERRα expression groups was analyzed using fow cytometry after 24 h of DDP treatment. KLE and HEC-1A cells treated with DDP for 24 h were used as the control group. **C** LDH activity of cell culture supernatants was measured in diferent ERRα expression groups after DDP treatment for 12 h. KLE and HEC-1A cells treated with DDP for 12 h were included as the control group in the experiment. **D** KLE and HEC-1A cells were treated with DDP at various concentration gradients for 48 h, and the IC_50_ of DDP in diferent ERRα expression groups was determined using the CCK8 assay. **E** Representative TEM images of KLE and HEC-1A cells in diferent ERRα expression groups treated with 7 µg DDP for 12 h. The mitochondrial membrane structure was indistinctly dissolved, the matrix was dissolved in a large area, and the crest was broken in siERRα cells, as indicated with the red arrows; the yellow arrows indicate ERRα-overexpressing EC cells whose mitochondrial membrane structure was relatively clear, the matrix was partially dissolved, and the cristae were slightly broken. The blue arrows indicate the mitochondria in the KLE and HEC-1A cells of control group treated with DDP for 12 h. Scale: 2 μm. **F** Basal, ATP-linked, and maximal respiration and spare respiratory capacities were assessed to evaluate the mitochondrial function in diferent ERRα expression groups. KLE^+DDP^ and HEC-1A^+DDP^ cells were used as the control groups. **G** Total RNA in KLE and HEC-1A cells was assessed using RT-qPCR to measure the expression of glycolysis-related genes in the ovERRα and siERRα groups. KLE and HEC-1A cells were used as the control group. H Extracellular acidifcation rate, glycolysis, glycolytic capacity, and glycolytic reserve in diferent ERRα expression groups are shown. KLE and HEC-1A cells were used as the control group. I Total RNA in KLE and HEC-1A cells was assessed using RT-qPCR to measure the expression of pyroptosis-related genes in EC cells. KLE^−ovERRα+DDP^ and HEC-1A^−ovERRα+DDP^ cells were used as the control groups. The results are presented as the average of three experimental replicates. Data are shown as the mean±SD. Statistical tests: Student’s *t*-test. *p<0.05; **p<0.01; ***p<0.001; ****p<0.0001. Abbreviations: EC, endometrial cancer; IC_50_, inhibitory concentration; PI, propidium iodide; TEM, transmission electron microscopy; 2-DG, 2-deoxy-glucose

The correction does not compromise the validity of the conclusions and the overall content of the article. The original article [1] has been updated.
