# Supplementary figures and images for: ERRα promotes glycolytic metabolism and targets the NLRP3/caspase-1/GSDMD pathway to regulate pyroptosis in endometrial cancer
Source: J Exp Clin Cancer Res. 2023 Oct 20;42:274. doi: 10.1186/s13046-023-02834-7 (PMC10588109; doi:10.1186/s13046-023-02834-7)

≤ ≥ ≪ ≫ zoom in 1.5x 3x 10x base zoom out 1.5x

**Table 1**

\_\_\_\_\_

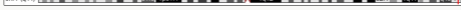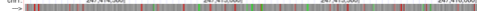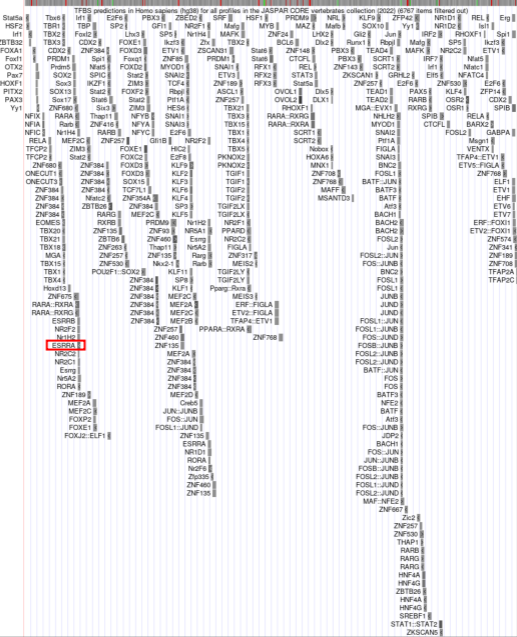

Supplement: Supplementary file 1 — Additional file 1. [file 13046_2023_2834_MOESM1_ESM.pdf]

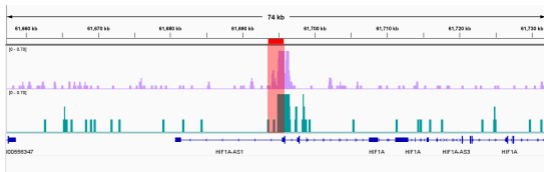

Supplement: Supplementary file 3 — Additional file 3. [file 13046_2023_2834_MOESM3_ESM.pdf]

**A**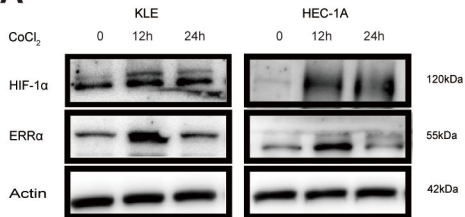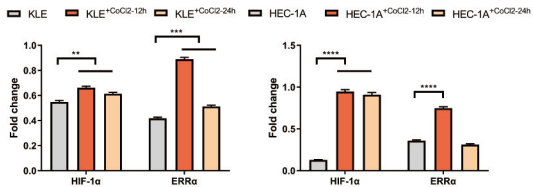**B**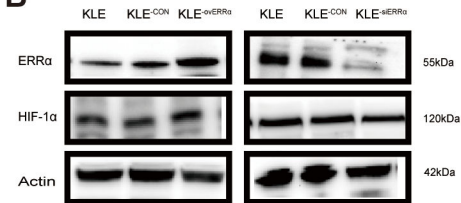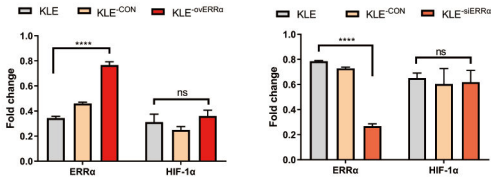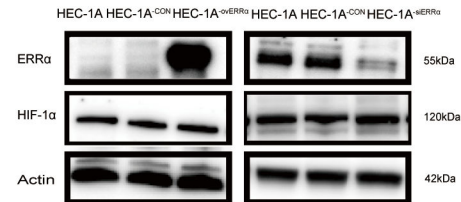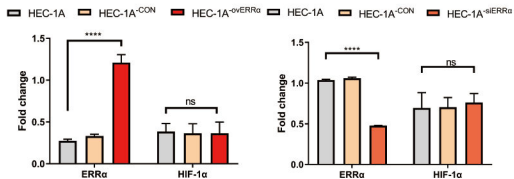

Supplement: Supplementary file 4 — Additional file 4. [file 13046_2023_2834_MOESM4_ESM.pdf]

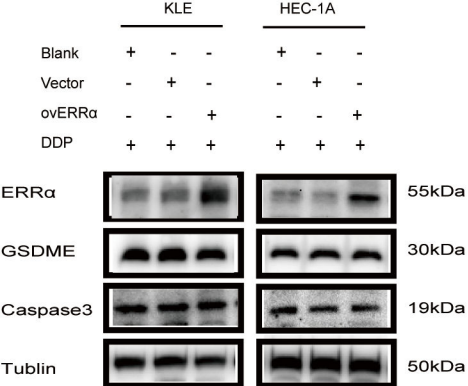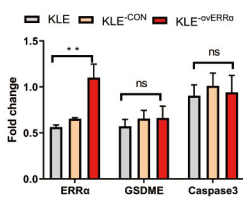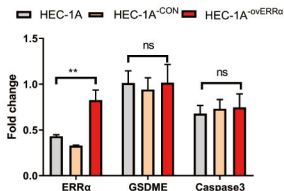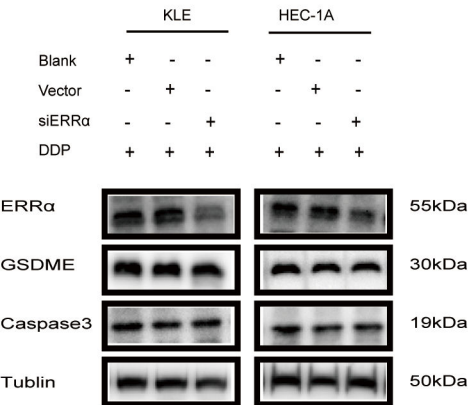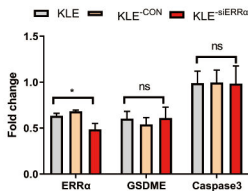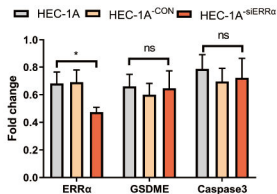

Supplement: Supplementary file 5 — Additional file 5. [file 13046_2023_2834_MOESM5_ESM.pdf]
