# Supplementary material for: ERRα promotes glycolytic metabolism and targets the NLRP3/caspase-1/GSDMD pathway to regulate pyroptosis in endometrial cancer
Source: J Exp Clin Cancer Res. 2023 Oct 20;42:274. doi: 10.1186/s13046-023-02834-7 (PMC10588109; doi:10.1186/s13046-023-02834-7)
Supplement: Supplementary file 2 — Additional file 2. [file 13046_2023_2834_MOESM2_ESM.pdf]

Showing 2.10kb region from base 247414077 to 247416176.

Homo sapiens chromosome 1, GRCh38.p14 Primary Assembly

NCBI Reference Sequence: NC_000001.11

GenBank Graphics

```
>NC_000001.11:247414077-247416176 Homo sapiens chromosome 1, GRCh38.p14 Primary Assembly
TATTTTCTTCTAGGAGTTTTACAGTTTTAGATCTTATGTTTAGATCTTTAATCCATTTTGAGTTGGTTTT
TGTGTGTGCCAAGTTTTATTCTTTTGCGTGTGGAAATCCAGTTTCACCAAAAGCTTAATTTTTCAAAATC
TTATATTTTTCTTGATTTTTTTTTCACACTTCTATAGCCAACATCTTTATTGAAGAGATTGTCCTTTCTC
CATTGTGTCTTCTTGGTGACCTTGTCAAAGAATTAGTTGACTGCATATACTTGAGTTTATTTCTGTTCCA
CTGGTCTATGGGTCTGTTTTTATAAAGGCATGGATTTTTACTGCATATGAATTATACCTAAATTTGTCTT
TTTTTTTTCCTTTTTCTGGAGAATGGGGTCTCACTATATTGCCCAGGCAGGTCTCGAACTCCTGGGCTCA
AGCTATCCTCCCGCCTCTTGCCTCCCTGAGAGCTGGGATTACAGGCGTGAGCCACTGCGCCCGGCTGAAT
TATACCTAAATTAACTTGACTAAACAAACAAAAACAAAAAGATCCCAGGTTCAGCCAATGAGCTCCTTTT
TTTTTTTTTGAAATGGAGTCTTGCTCTTGTCACCCAGGCTGGAGTGCAGTGGCACAATCTCAGCTCACTG
CAGCCTCCACCTCCCAGGTTCAAGTGATTCTCTTGCCTCAGCTTCCTGAGTAGCTGGGATTACAGGTGCC
CGCCACCACACCCAGCTATTTTTTTTTATATTTTTAGTAGAGATGGGGTTTCGTCATGTTGGCCAGGCTG
GTCTCAAACTCCTGACCTTGTGATCTGCCTGCCTTGGCCTCTCAAAGTGCTGGGATTACAGGCATGAGCC
ACTGCACCCAGGCTGAGCTCCTTTCTTTTGGAGCATATTTGGTCTGCTTTCTCTCTAGCTTCAGCACCTG
AAGTTTTTGCTGCTCCTGCCTCAGCTCCAGCCCCGAATGACACCTTTCACCTGGTTCATGGAGACTCAGG
TCTGCTGTCATGCCCCTAAGCAGGACTAGATGCCTCTCTCATGTTGTAGAGCATTCTCTCTGCAGTTCTG
TAGAAAGGTGGTAGTCATCGGAACCAACTGCTTATTCTAGCTTCTCTGTGCCAGGCTCTGTTTTAAAGGC
CTCACGTTTAGCCATTCACTCAGTGTTTTCTGACAGCCTTCTAGGATAGGTACCGTTATTTGGGTTGATG
TAGTGCTTGGTCAAATGCAGTATAATGAAGGGATCAAGGAGGGGGCAATTCCAAGCAAAACCACTTGTTC
ATCCATCCTGAGTCTAGGAGATTTGGAAGATCTAAAGAAGTAGCAGGCAATTATTGTCACCCTTTTACAG
GTATGAGGCACAGACAGACCCAGTCATTTGCTGACATCCCTCCACTGAACTGGGTGCTCGTCCTTTTCAC
TCACCCAGAGGCTGAGCGAGTGCCTCTCACCCACCAAGCTCTGTTGAACTTGTGCCTTTGCCGGTCTGTG
GTCCTGCGGCCGTCTCATGAGCTGCAGAGTAGGTCTGTGTGTGTCTCTCCTCAAGCTACTCAAGCTGAGG
CTTTGTGTGCATGCCTGCCACATACCAGCCATTCCGTGAGTGTTAGTGGAACAGGTGAGTCAATGAGTCA
GGGAGTAAAATTTGTGCTTTAAAAGTAGAACATGTCGGAGAGAACCTGTACTGCCTTCCAGCCATCTTGC
TGTGAGAGGACAGGGCATGGACTCTGGAGTTTGAAGGCTGGGTCAGGTCATGCTCTGGATTCTTTACTCA
CTCGCATGGCATGTCCTTAGTTTCACTTCCCTCACTCTCAGTGGAGCCGTTGCAGCGGGCAGTGTCAGAG
GCAACATCCATTTAGCACTGCCGATTTGACCCCCAGCCCCATCTCATACTGGCCTCCCACCCTGCTTCTG
TGGGGTGCCTCAGAGGAGAGCAAGGTGAACCCCAGTATGGAACCGAGACACGGTTTTGACAACACTGTTG
ATCCCATTTGGAAAATCCAGCTATGTGATTAACCATTAGTCTCTCTGCCTCTGCTCTGATGTAAGTGGAG
ACCACATCCTTCCTGCCCCTTCTGGGGCTGCGACTGCTATAAATTCATTGCATTTCCTCTCTAGCTGTTC
```
