# Supplementary material for: ERRα promotes glycolytic metabolism and targets the NLRP3/caspase-1/GSDMD pathway to regulate pyroptosis in endometrial cancer
Source: J Exp Clin Cancer Res. 2023 Oct 20;42:274. doi: 10.1186/s13046-023-02834-7 (PMC10588109; doi:10.1186/s13046-023-02834-7)
Supplement: Supplementary file 6 — Additional file 6. [file 13046_2023_2834_MOESM6_ESM.pdf]

Supplement Table 1. Study on the clinicopathological information of endometrial cancer organoids

Identifier	Age (year)	Pathology type	FIGO Stage	Tumor grade	Depth of myometrial invasion	Lymphovascular space invasion	Lymphatic metastasis
EC4	57	Endometrioid adenocarcinoma	IA	II	<1/2	-	-
EC5	54	Endometrioid adenocarcinoma	IIIA	III	≥1/2	+	-
EC6	66	Endometrioid adenocarcinoma	IA	I	<1/2	-	-
EC8	51	Endometrioid adenocarcinoma	IA	II	<1/2	+	-
EC9	58	Endometrioid adenocarcinoma	IA	II	<1/2	+	-
EC10	61	Endometrioid adenocarcinoma	IA	III	<1/2	+	-
EC11	67	Endometrioid adenocarcinoma	IB	I	≥1/2	+	-
EC12	45	Endometrioid adenocarcinoma	IA	II	<1/2	-	-
EC13	53	Endometrioid adenocarcinoma	IA	II	<1/2	-	-
EC14	58	Endometrioid adenocarcinoma	IA	II	<1/2	-	-
EC16	62	Endometrioid adenocarcinoma	IA	III	<1/2	-	-
