# Supplementary material for: ERRα promotes glycolytic metabolism and targets the NLRP3/caspase-1/GSDMD pathway to regulate pyroptosis in endometrial cancer
Source: J Exp Clin Cancer Res. 2023 Oct 20;42:274. doi: 10.1186/s13046-023-02834-7 (PMC10588109; doi:10.1186/s13046-023-02834-7)
Supplement: Supplementary file 7 — Additional file 7. [file 13046_2023_2834_MOESM7_ESM.pdf]

Supplement Table 2. ChIPseeker annotations on the target gene

Chromosome	annotation	geneStart	geneEnd	geneLength	geneStrand	geneId	transcriptId	geneName	geneDescription
14	Promoter (<=1kb)	61695513	61721824	26312	+	ENSG00000100644	ENST00000557446	HIF1A	hypoxia inducible factor 1 subunit alpha [Source:HGNC Symbol;Acc:HGNC:4910] && Q16665.1 RecName: Full=Hypoxia-inducible factor 1-alpha; Short=HIF-1-alpha; Short=HIF1-alpha; AltName: Full=ARNT-interacting protein; AltName: Full=Basic-helix-loop-helix-PAS protein MOP1; AltName: Full=Class E basic helix-loop-helix protein 78; Short=bHLHe78; AltName: Full=Member of PAS protein 1; AltName: Full=PAS domain-containing protein 8
1	Promoter (<=1kb)	247416156	247449102	32947	+	ENSG00000162711	ENST00000366497	NLRP3	NLR family pyrin domain containing 3 [Source:HGNC Symbol;Acc:HGNC:16400] && Q96P20.3 RecName: Full=NACHT, LRR and PYD domains-containing protein 3; AltName: Full=Angiotensin/vasopressin receptor AII/AVP-like; AltName: Full=Caterpiller protein 1.1; Short=CLR1.1; AltName: Full=Cold-induced autoinflammatory syndrome 1 protein; AltName: Full=Cryopyrin; AltName: Full=PYRIN-containing APAF1-like protein 1
