# Supplementary material for: ERRα promotes glycolytic metabolism and targets the NLRP3/caspase-1/GSDMD pathway to regulate pyroptosis in endometrial cancer
Source: J Exp Clin Cancer Res. 2023 Oct 20;42:274. doi: 10.1186/s13046-023-02834-7 (PMC10588109; doi:10.1186/s13046-023-02834-7)
Supplement: Supplementary file 8 — Additional file 8. [file 13046_2023_2834_MOESM8_ESM.pdf]

Supplement Table 3. Correlation analysis between ERRα expression and clinicopathological characteristics of patients in TCGA database

Characteristic	Low expression of ERRα<br>276	High expression of ERRα<br>276	P
**Age, median (IQR)**	64 (57, 72)	64 (57, 71)	0.542
**Age, n (%)**			0.817
<=60	105 (19.1%)	101 (18.4%)	
>60	170 (31%)	173 (31.5%)	
**BMI, n (%)**			0.687
<=30	111 (21.4%)	101 (19.5%)	
>30	154 (29.7%)	153 (29.5%)	
**Race, n (%)**			*0.003*
Asian	12 (2.4%)	8 (1.6%)	
Black or African American	38 (7.5%)	70 (13.8%)	
White	202 (39.8%)	177 (34.9%)	
**Diabetes, n (%)**			0.108
No	157 (34.8%)	171 (37.9%)	
Yes	70 (15.5%)	53 (11.8%)	
**Menopause status, n (%)**			0.957
Pre	18 (3.6%)	17 (3.4%)	
Peri	8 (1.6%)	9 (1.8%)	
Post	226 (44.7%)	228 (45.1%)	
**Hormones therapy, n (%)**			0.260
No	147 (42.7%)	150 (43.6%)	
Yes	28 (8.1%)	19 (5.5%)	
**Histological type, n (%)**			0.173
Endometrioid	214 (38.8%)	196 (35.5%)	
Mixed	9 (1.6%)	15 (2.7%)	
Serous	53 (9.6%)	65 (11.8%)	
**Clinical stage, n (%)**			0.065
Stage I	185 (33.5%)	157 (28.4%)	
Stage II	24 (4.3%)	27 (4.9%)	
Stage III	57 (10.3%)	73 (13.2%)	
Stage IV	10 (1.8%)	19 (3.4%)	
**Histologic grade, n (%)**			*0.002*
G1	64 (11.8%)	34 (6.3%)	
G2	62 (11.5%)	58 (10.7%)	
G3	146 (27%)	177 (32.7%)	
**Tumor invasion(%), n (%)**			0.339
<50	126 (26.6%)	133 (28.1%)	
>=50	115 (24.3%)	100 (21.1%)	
**Surgical approach, n (%)**			0.235
Minimally Invasive	98 (18.5%)	110 (20.8%)	
open	170 (32.1%)	152 (28.7%)	

**Radiation therapy, n (%)**			*0.030*
No	158 (30%)	121 (23%)	
Yes	116 (22%)	132 (25%)	
**Primary therapy outcome, n (%)**			0.599
PD	8 (1.7%)	12 (2.5%)	
SD	2 (0.4%)	4 (0.8%)	
PR	7 (1.5%)	5 (1%)	
CR	226 (47.1%)	216 (45%)	
**Residual tumor, n (%)**			*0.010*
R0	184 (44.6%)	191 (46.2%)	
R1	18 (4.4%)	4 (1%)	
R2	7 (1.7%)	9 (2.2%)	

Footnotes: 552 primary EC cases with clinical information and gene expression data were retrieved from TCGA database. The parametric P<0.05 (in italic) was considered to be statistically significant.

Abbreviations: TCGA, The Cancer Genome Atlas; BMI, Body mass index; IQR, Interquartile range; PD, Progressive disease; SD, Stable disease; PR, Partial response; CR, Complete response.
