# Supplementary material for: ERRα promotes glycolytic metabolism and targets the NLRP3/caspase-1/GSDMD pathway to regulate pyroptosis in endometrial cancer
Source: J Exp Clin Cancer Res. 2023 Oct 20;42:274. doi: 10.1186/s13046-023-02834-7 (PMC10588109; doi:10.1186/s13046-023-02834-7)
Supplement: Supplementary file 9 — Additional file 9. [file 13046_2023_2834_MOESM9_ESM.pdf]

Supplement Table 4. Univariate and multivariate analyses of overall survival in patients with EC from TCGA

Characteristics	Total (N)	HR(95% CI) Univariate analysis	P value	HR(95% CI) Multivariate analysis	P value
**Age**	551				
<= 60	207	Reference		Reference	
> 60	344	1.850 (1.162 - 2.944)	*0.009*	1.254 (0.606 - 2.597)	0.541
**BMI**	520				
<= 30	211	Reference			
> 30	309	0.963 (0.634 - 1.464)	0.861		
**Race**	508				
Asian	20	Reference			
Black or Afro American&White	488	2.888 (0.710 - 11.757)	0.139		
**Diabetes**	453				
No	329	Reference			
Yes	124	1.167 (0.728 - 1.871)	0.520		
**Menopause status**	506				
Pre	35	Reference			
Peri&Post	471	0.827 (0.381 - 1.791)	0.629		
**Hormones therapy**	346				
**No**	299	Reference			
Yes	47	0.804 (0.381 - 1.695)	0.566		
**Histological type**	553				
Endometrioid	411	Reference		Reference	
Mixed&Serous	142	2.636 (1.751 - 3.968)	< *0.001*	1.458 (0.709 - 2.996)	0.305
**Clinical stage**	553				
Stage I	342	Reference		Reference	
Stage II&Stage III&Stage IV	211	3.267 (2.144 - 4.980)	< *0.001*	2.291 (1.090 - 4.813)	*0.029*
**Histologic grade**	542				
G1	99	Reference		Reference	
G2&G3	443	11.641 (2.864 - 47.318)	< *0.001*	10.432 (1.381 - 78.774)	*0.023*
**Tumor invasion(%)**	475				
< 50	261	Reference		Reference	
>= 50	214	2.825 (1.752 - 4.554)	< *0.001*	1.494 (0.746 - 2.992)	0.257
**Radiation therapy**	529				
No	281	Reference		Reference	
Yes	248	0.596 (0.387 - 0.919)	*0.019*	0.289 (0.151 - 0.555)	< *0.001*
**Primary**	482				

**therapy outcome**					
SD&PR&CR	462	Reference		Reference	
PD	20	7.844 (4.279 - 14.376)	*< 0.001*	3.373 (1.047 - 10.862)	*0.042*
**Residual tumor**	414				
R0	376	Reference		Reference	
R1&R2	38	3.112 (1.774 - 5.459)	*< 0.001*	2.807 (1.134 - 6.952)	*0.026*

Footnotes: The parametric P<0.05 (in italic) was considered to be statistically significant.

Abbreviations: EC, Endometrial cancer; TCGA, The Cancer Genome Atlas; BMI, Body mass index; IQR, Interquartile range; PD, Progressive disease; SD, Stable disease; PR, Partial response; CR, Complete response.
